# Reciprocal expression of Annexin A6 and RasGRF2 discriminates rapidly growing from invasive triple negative breast cancer subsets

**DOI:** 10.1371/journal.pone.0231711

**Published:** 2020-04-16

**Authors:** Olga Y. Korolkova, Sarrah E. Widatalla, Diva S. Whalen, Gladys N. Nangami, Adeniyi Abimbola, Stephen D. Williams, Heather K. Beasley, Emily Reisenbichler, Mary Kay Washington, Josiah Ochieng, Ingrid A. Mayer, Brian D. Lehmann, Amos M. Sakwe

**Affiliations:** 1 Department of Biochemistry, Cancer Biology, Neuroscience and Pharmacology, School of Graduate Studies and Research, Meharry Medical College, Nashville, Tennessee, United States of America; 2 Department of Pathology, Yale Medical School, New Haven, Connecticut, United States of America; 3 Department of Pathology, Vanderbilt University Medical Center, Nashville, Tennessee, United States of America; 4 Department of Medicine, Vanderbilt University Medical Center, Nashville, Tennessee, United States of America; University of Kansas Medical Center and VA Medical Center, UNITED STATES

## Abstract

Actively growing tumors are often histologically associated with Ki67 positivity, while the detection of invasiveness relies on non-quantitative pathologic evaluation of mostly advanced tumors. We recently reported that reduced expression of the Ca^2+^-dependent membrane-binding annexin A6 (AnxA6) is associated with increased expression of the Ca^2+^ activated RasGRF2 (GRF2), and that the expression status of these proteins inversely influence the growth and motility of triple negative breast cancer (TNBC) cells. Here, we establish that the reciprocal expression of AnxA6 and GRF2 is at least in part, dependent on inhibition of non-selective Ca^2+^ channels in AnxA6-low but not AnxA6-high TNBC cells. Immunohistochemical staining of breast cancer tissues revealed that compared to non-TNBC tumors, TNBC tumors express lower levels of AnxA6 and higher Ki67 expression. GRF2 expression levels strongly correlated with high Ki67 in pretreatment biopsies from patients with residual disease and with residual tumor size following chemotherapy. Elevated AnxA6 expression more reliably identified patients who responded to chemotherapy, while low AnxA6 levels were significantly associated with shorter distant relapse-free survival. Finally, the reciprocal expression of AnxA6 and GRF2 can delineate GRF2-low/AnxA6-high invasive from GRF2-high/AnxA6-low rapidly growing TNBCs. These data suggest that AnxA6 may be a reliable biomarker for distant relapse-free survival and response of TNBC patients to chemotherapy, and that the reciprocal expression of AnxA6 and GRF2 can reliably delineate TNBCs into rapidly growing and invasive subsets which may be more relevant for subset-specific therapeutic interventions.

## Introduction

Triple negative breast cancer (TNBC) represents approximately 20% of all diagnosed breast cancer patients, but accounts for significantly higher (>80%) breast cancer associated mortality. This is attributed in part, to the frequent relapse of more aggressive and/or metastatic tumors especially after therapeutic interventions [1–3]. This notwithstanding, TNBC comprises a diverse array of phenotypes and this heterogeneity is believed to account for the diverse and often poor responses to chemotherapy, targeted therapies and combinations of these agents. Thus far, four distinct molecular subtypes, including the immune active (BL1/BLIA), the immune suppressed (BL2/BLIS), the immune devoid mesenchymal-like (MES) and the luminal androgen receptor positive (LAR) subtypes [4–6], have been characterized and demonstrated to be associated with distinct responses to therapies and distinct patient outcomes. However, the use of these categories of TNBC in the design of treatment options for individual patients remains untested and challenging.

Other classifications e.g. those based on cell morphology as either basal-like or mesenchymal-like [7], extent of genomic instability [8], and expression of phenotypic markers such as vimentin (mesenchymal), E-cadherin (epithelial) and cytokeratins also reveal significant variability among TNBCs [9]. For instance, the expression of epithelial and mesenchymal markers depends on the stage of the tumor with respect to the epithelial-to-mesenchymal transition (EMT) or the reverse process mesenchymal-to-epithelial transition [10, 11]. On the other hand, pathological evaluations often classify TNBC tumors into those that grow rapidly, those that grow poorly but highly invasive and those that neither grow rapidly nor are invasive (indolent). Interestingly, actively growing TNBCs are histologically associated with high mitotic indices or positivity for proliferating cell markers such as Ki67, detection of tumor invasiveness remains dependent on pathologic evaluation of mostly high grade or advanced tumors.

Several studies have shown that the calcium dependent membrane binding Annexin A6 (AnxA6) is downregulated in malignant forms of breast cancer [12], gastric cancer [13] melanomas [14], esophageal adenocarcinoma [15] and several other solid tumors [16]. As a Ca^2+^ and membrane binding protein, AnxA6 is implicated in a wide range of cellular functions including cell growth, differentiation and motility which underlie tumor progression. Therefore, reduced expression or loss of AnxA6 is associated with decreased cell motility, early onset and rapid growth of xenograft TNBC tumors *in vivo* [17]. This decrease in the expression of AnxA6 has now been reported to affect several key cellular processes including Ca^2+^ homeostasis, cholesterol homeostasis, energy metabolism, cell surface receptor mediated signaling, focal adhesions, vesicular transport, exocytosis and endocytosis, membrane repair as well as cell-cell and cell-extracellular matrix interactions [17]. We have also shown that reduced expression of AnxA6 in TNBC cells is associated with increased expression of RasGRF2 (GRF2), a Ca^2+^ activated Ras protein specific guanine nucleotide exchange factor (RasGEF) [17], with little effect on other RasGEFs such as SOS1. Meanwhile, GRF2 has been reported to promote cell growth via activation of Ras proteins and to inhibit cell motility via interaction with Cdc42 and Rac1 Rho GTPases [18, 19]. Together, these studies suggest that the reciprocal expression of AnxA6 and GRF2 underlies the potential for TNBC cells to either grow rapidly or to become invasive.

In this study, we hypothesized that the reciprocal expression of AnxA6 and GRF2 is dependent on inhibition of non-selective Ca^2+^ channels and can be used to delineate potentially rapidly growing from invasive TNBCs. To test this, we assessed the reciprocal expression of AnxA6 and GRF2 in TNBC cells following treatment of cells with non-selective Ca^2+^ channel blockers. We also analyzed the expression of AnxA6, GRF2, SOS1 and Ki67 by immunohistochemistry (IHC) in a breast disease spectrum and following chemotherapy. Finally, we evaluated the potential for the reciprocal expression of AnxA6 and GRF2 to delineate invasive from rapidly growing TNBC cell lines and PDX models. Our data suggest that the reciprocal expression of AnxA6 and GRF2 may be useful to clearly delineate potentially proliferative GRF2-high/AnxA6-low mostly basal-like TNBCs from highly invasive GRF2-low/AnxA6-high mostly mesenchymal-like TNBC subsets. Our data also suggest the potential for AnxA6 expression status to independently predict distant relapse-free survival and overall response of TNBC patients to chemotherapy.

## Materials and methods

### Clinical and pathological features of study samples

The study reported here was approved by the Meharry Medical College Institutional Review Board (IRB) as exempt research (IRB # FWA00003675). A breast disease spectrum tissue microarray (TMA) comprising 192 cases/cores was purchased from US Biomax Inc. (Cat# BR2082b). The TMA contained 32 cases of metastatic carcinoma (lymph node), 69 invasive ductal carcinoma, 21 lobular carcinoma, 4 squamous cell carcinoma, 17 intraductal carcinoma, 1 lobular carcinoma in situ, 9 fibroadenoma, 8 hyperplasia, 12 inflammation (which included 4 plasma cell mastitis, 6 chronic mastitis, and 2 ductal ectasia with acute attack), 17 adjacent normal breast tissue and 2 normal breast tissue. Provided for each tissue was information on the TNM clinical stage, pathology grade as well as the expression status of ER, PR, HER-2 https://www.biomax.us/tissue-arrays/Breast/BR2082b.

Formalin-fixed paraffin-embedded (FFPE) biopsies and chemotherapy resistant residual de-identified tissues from 22 patients diagnosed with high grade TNBC were obtained from Vanderbilt University Medical Center. The patients received a variety of cytotoxic drugs including doxorubicin (adriamycin), cyclophosphamide (cytoxan), platinum-based cytotoxic compounds (cisplatin, carboplatin), abraxane (nab-paclitaxel), paclitaxel (taxol), capecitabine (xeloda), alone or in combination for variable periods. For each patient, 4 μm sections of the FFPE biopsy of the primary tumor and where applicable, the residual tumor were prepared on microscope slides. The clinical and pathological characteristics, the neoadjuvant chemotherapy regimen received by each patient and the size of the residual tumors are summarized in Table 1.

**Table 1:** Characteristics of biopsy and residual triple negative breast tumor samples.

BIOPSY #	Neoadjuvant Treatments	Residual Tumors/size
**1**	T, dd AC	NRT
**2**	T + Cisp	NRT
**3**	T, AC	RT—1.6 mm
**4**	dd AC + T	RT—2 mm
**5**	T + Carb, AC	NRT
**6**	dd AC + Abrax	RT—26 mm
**7**	AC and T	RT—27 mm
**8**	T + Cisp	NRT
**9**	AC + T	RT—35 mm
**10**	dd AC-T	RT—0.5 mm
**11**	dd AC-T	RT—7 and 5 mm foci
**12**	Cisp, T, AC	RT—14 mm
**13**	Cisp, AC	NRT
**14**	Abrax, AC	NRT
**15**	dd AC, T/Carb	NRT/NA
**16**	dd AC-T	RT—3 mm
**17**	Xeeloda	NM
**18**	dd AC-T	NRT
**19**	BRE1440, Cisp	RT—25 mm
**20**	dd AC-T	NRT
**21**	T, AC	RT—37 & 17 mm foci
**22**	T, AC	RT—7 mm

**NRT** = No Residual Tumor; **RT** = Residual Tumor; **NM** = No mastectomy; **NA** = No tumor sample available; **T** = Taxol; **AC** = Adriamycin and Cytoxan; **Cisp** = Cisplatin; **Carb** = Carboplatin; **dd AC** = dose-dense Adriamycin; **Abrax** = Abraxane; **Xeloda** = Capecitabine; BRE1440

We also used a publicly available Gene Expression Omnibus (GEO) dataset (accession number GSE25065) with data from 64 TNBC and 132 non-TNBC patients who underwent neoadjuvant taxane and anthracycline-based chemotherapy. Detailed characteristics of the patient and biospecimen cohorts, as well as the types of biopsies collected for genomic analysis and the chemotherapy regimens administered are described in Hatzis et al., 2011 [20]. In this dataset, 165 of the 196 patients (84%) received neoadjuvant chemotherapy. Finally, we used the Receiver Operating Characteristic (ROC) plotter for breast cancer (www.rocplot.org) which links transcriptomic gene expression profiles of 3,104 breast cancer patients to their response to therapy, [21] to determine the likelihood of response to chemotherapy based on the expression status of our biomarkers in TNBC tissues. Responder and non-responder patients were compared using Mann-Whitney test or ROC test in the R statistical package.

### Immunohistochemistry

Thin (4 μm) tissue sections on microscope slides and the TMAs were stained with antibodies against AnxA6 (Santa Cruz, sc-1931), GRF2 (Abcam, ab121577), SOS1 (Sc-10803), Ki67 (Vector Laboratories, VP-K451) as well as EGFR (Santa Cruz, sc-373746), p-EGFR (Invitrogen, Cat# 44-794G) and p-ERK1/2 (Abcam, ab47310), at the Vanderbilt Translational Pathology Shared Resource (https://ww2.mc.vanderbilt.edu/tpsr/). Positive controls were human placenta tissue for AnxA6, human tonsil tissue for GRF2 and Ki67, human breast tissue for SOS1 and p-EK1/2, and mutant EGFR lung adenocarcinoma xenograft tumor for EGFR and p-EGFR.

High resolution scanning of whole slides using a Leica SCN400 slide scanner (Leica Biosystems) at 20x to a resolution of 0.5 μm/pixel was performed at the Digital Histology Shared Resource at Vanderbilt University Medical Center (www.mc.vanderbilt.edu/dhsr). All images were captured at a 20x magnification and quantification of immunostaining of the tumor areas (staining intensity) was performed using the Tissue IA, v2.0 software (Leica Microsystems) which consists of algorithms for unbiased, automated image analysis. Briefly, color deconvolution for 3,3’-diaminobenzidine (DAB) staining was applied, and the threshold was set for differentiating tumor tissue from the background. The tumor tissue region(s) on each slide were manually selected and the DAB staining intensity analyzed. When multiple regions were analyzed within the same tissue section, the average staining intensity was determined and used in subsequent analyses.

### Cell transfection, treatment with Ca2+ channel blockers and western blotting

The breast epithelial MCF-10A and the TNBC cell lines including BT-549, HCC1806, MDA-MB-231 and MDA-MB-468 TNBC cells were purchased from ATCC, East Rutherford, NJ. BT-549, HCC1806 and MDA-MB-231 cells were maintained in DMEM/F12 while MDA-MB-468 were cultured in Leibovitz's L-15 Medium (ThermoFisher) supplemented with 0.15% NaHCO_3._ These and other cell lines in our collection were authenticated in October 2018 and only early passages of the authenticated cell lines were used in our studies. All media were also supplemented with 10% fetal bovine serum (FBS,), L-glutamine (2 mM), 100 units/ml penicillin, and 50 units/ml streptomycin (Invitrogen, Carlsbad, CA). HCC1806 cells stably expressing AnxA6 (1806-Anx6) or empty vector control (1806-EV) were generated as previously described [22]. These cell lines were cultured in a humidified 95% air and 5% CO_2_ incubator at 37° C and media were changed every 2–3 days.

For treatment of cells with calcium channel blockers (CCBs), nickel sulfate (Ni^2+^) was reconstituted in complete medium, while bepridil and amlodipine were reconstituted in dimethyl sulfoxide (DMSO). Cells were plated and allowed to attach overnight, then treated with the indicated concentrations of these drugs or the vehicle for the indicated times. Cells were harvested and used for western blotting as described below.

For Western blotting, TNBC cells with or without the indicated treatments were lysed in RIPA buffer (50mM Tris-HCl, 150mM NaCl, 1mM EDTA, 20mM NaF, 5 mM Na_3_VO4, 0.25% sodium deoxycholate, 1% Triton X-100) and freshly added protease inhibitor cocktail (Sigma). Whole cell extracts of PDX models (n = 19) in RIPA buffer were kindly donated by Dr. Jennifer Pietenpol, Vanderbilt Ingram Cancer Center. Equal amounts of the cleared lysates (30 μg) were subjected to 4–12% SDS-PAGE, transferred to nitrocellulose membrane and probed with the indicated antibodies. Blots were revealed by enhanced chemiluminescence (Perkin Elmer).

### Statistical analysis

The relationship between differential biomarker expression and relapse-free survival of breast cancer patients, was carried out by using raw data from a publicly available database from Gene Expression Omnibus (GEO) with accession number GSE25065 [20]. This dataset contains data on 196 breast cancer patients who underwent neoadjuvant taxane and anthracycline-based chemotherapy treatment, and includes information on their time to distant relapse-free survival (DRFS). Kaplan-Meier survival plots and log-rank tests (https://www.evanmiller.org/ab-testing/survival-curves.html) were used to assess any differences in the time to DRFS for each marker. For all other analyses, the Mann-Whitney test was used to compare subgroups and Pearson correlations were used to determine the relationship between subgroups. ROC test was used to determine the reliability of each protein as a biomarker. All other statistical analyses were performed using the IBM SPSS statistical software, version 25 (SPSS, Inc., Chicago, IL). Differences were considered statistically significant at p<0.05.

## Results

### Reciprocal regulation of AnxA6 and GRF2 by non-selective calcium channel blockers

**Fig 1:**
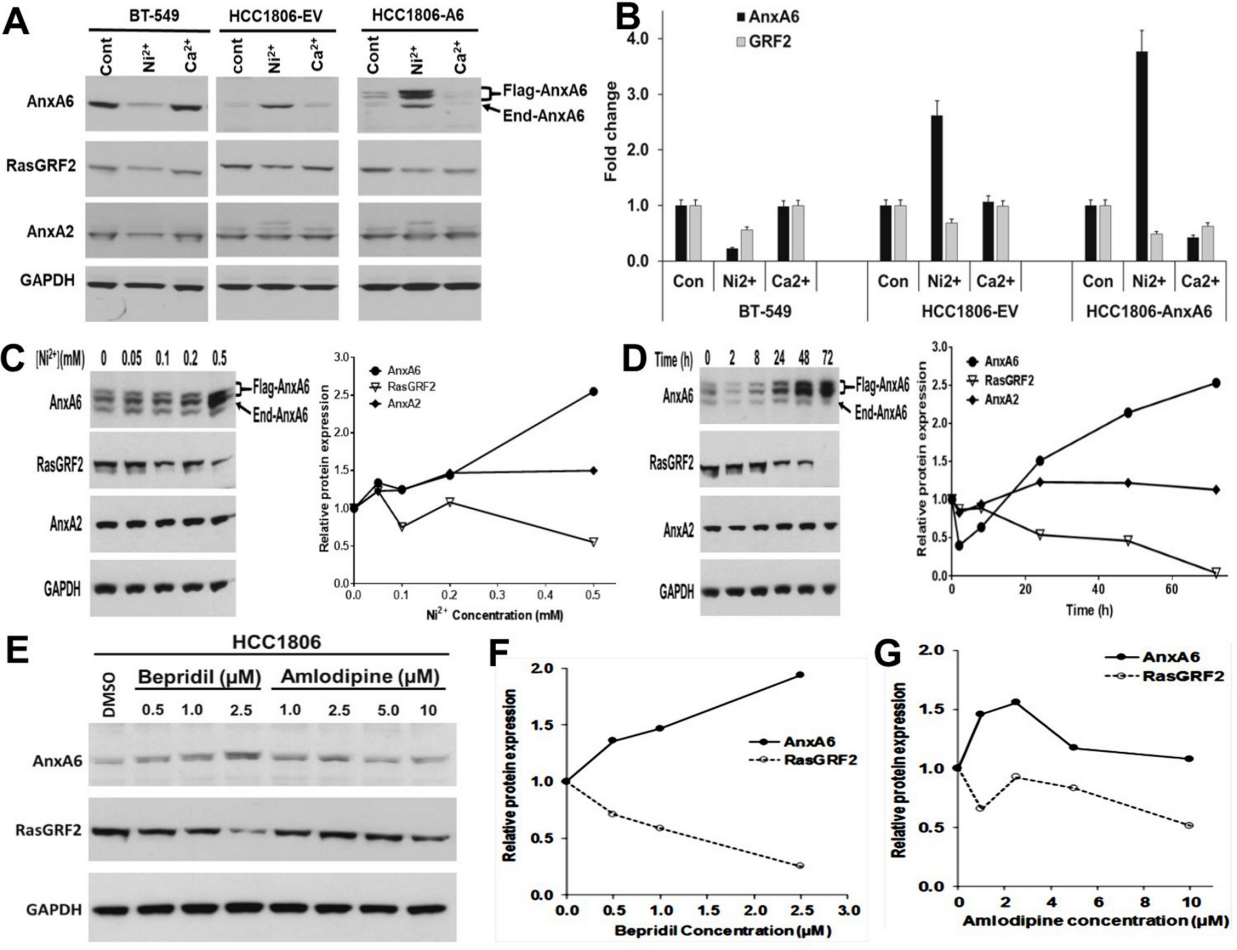
Reciprocal regulation of AnxA6 and GRF2 by non-selective calcium channel blockers. A) BT-549 (AnxA6 high) and empty vector or AnxA6 expressing HCC1806 (AnxA6-low) cells were treated with Ni^2+^ or Ca^2+^ for 72 h and AnxA6, AnxA2 and GRF2 protein assessed by immunoblotting. B) Densitometric analysis of AnxA6 and GRF2 expression. Bars represent fold change relative to control cells from two independent experiments. C and D) Effects of Ni^2+^ concentration and time on the reciprocal expression of AnxA6 and GRF2. AnxA6 expressing HCC1806 cells were treated with the indicated concentrations of Ni^2+^ for 72 h (C) or at 0.5 mM Ni^2+^ for the indicated times (D); Densitometric analysis of the expression of AnxA6, GRF2 and AnxA2 from a representative experiment (C and D, left panels). E) HCC1806 cells were treated with the indicated concentrations of bepridil or amlodipine for 72 h and the expression of AnxA6 and GRF2 assessed by western blotting. F and G) Densitometric analysis of the protein bands in bepridil treated (F) and amlodipine treated cells from a representative experiment (G). Flag-AnxA6: flag-tagged recombinant AnxA6, End-AnxA6: endogenously expressed AnxA6.

In a recent report we showed that activation of the Ca^2+^ mobilizing EGFR or stimulation of intracellular Ca^2+^ overload by treatment of cells with ionomycin, led to activation and rapid degradation of GRF2 in TNBC cells [17]. This effect was transient (<5 min) in the AnxA6-high BT-549 and MDA-MB-231 (MDA-231) cells but lasted longer (>10 min) in the AnxA6-low MDA-MB-468 (MDA-468) cells. We also showed that the Ca^2+^ induced degradation of GRF2 was blocked by either AnxA6 expression or chelation of extracellular Ca^2+^ with EGTA [17]. Although this supports the modulation of the cellular levels of GRF2 by Ca^2+^ influx and indirectly by AnxA6, this transient decrease in GRF2 levels is insufficient to explain the reciprocal expression of AnxA6 and GRF2 in TNBC cells. To verify that GRF2 and AnxA6 are mutually regulated in TNBC, we speculated that inhibition of Ca^2+^ influx may differentially affect the expression of these proteins in AnxA6-high mesenchymal-like and AnxA6-low basal-like TNBC cells. To test this, we treated TNBC cells that express relatively high or low AnxA6 [17, 22] with Ni^2+^, a non-selective Ca^2+^ channel blocker (CCB) [23] or high Ca^2+^ (3 mM). Treatment of AnxA6-high BT-549 TNBC cells with Ni^2+^ led to a decrease in both AnxA6 and GRF2 protein levels, while Ca^2+^ treatment had little or no effect (Fig 1A and 1B). On the contrary, treatment of the AnxA6-low HCC1806 TNBC cells (HCC1806-EV) or the AnxA6 expressing sub-clone of these cells (HCC1806-AnxA6) with Ni^2+^ led to AnxA6 up regulation (>2.5 fold) and a corresponding down regulation of GRF2. Using HCC1806-AnxA6 cells, we also show that the reciprocal expression of AnxA6 and GRF2 is Ni^2+^ concentration (Fig 1C) and time dependent (Fig 1D). AnxA6 can be expressed as two bands (64 and 68 kDa) due to alternative usage of two initiation codons [24]. In addition to the endogenous (End) AnxA6 band, the additional AnxA6 bands in the blots showing the expression of recombinant AnxA6 driven by a CMV promoter (Fig 1A, 1C and 1D and S1A Fig), are the two alternatively expressed AnxA6 species (Flag-AnxA6). These additional AnxA6 bands were also detected when AnxA6 was expressed in MDA-MB-468 TNBC cells [17] and are therefore, not degradation products.

We next determined whether other CCBs can elicit the reciprocal expression of AnxA6 and GRF2 in TNBC cells. Treatment of HCC1806 cells with bepridil, a non-selective CCB [25] led to increased AnxA6 and decreased RasGRF2 expression in a concentration dependent manner (Fig 1E and 1F), while treatment with amlodipine, an L-Type CCB [26] did not consistently lead to the reciprocal expression of AnxA6 and GRF2 (Fig 1E and 1G). As demonstrated in S1 Fig, the reciprocal expression of AnxA6 and GRF2 was replicated in the AnxA6-low breast epithelial MCF10A cells following treatment with Ni^2+^ (S1A Fig) and in the AnxA6-low MDA-468 TNBC cells following treatment with Ni^2+^ or bepridil but not amlodipine (S1B Fig). On the contrary, treatment of the AnxA6 high MDA-MB-231 cells did not elicit the reciprocal expression of AnxA6 and GRF2 (S1B Fig). These data suggest that although the expression of AnxA6 and GRF2 is inversely related in most TNBC cells, the expression of these proteins is mutually regulated only in the subset of TNBC cells expressing relatively low levels of AnxA6.

### Association of AnxA6 and GRF2 expression status with breast cancer progression and metastasis

**Fig 2:**
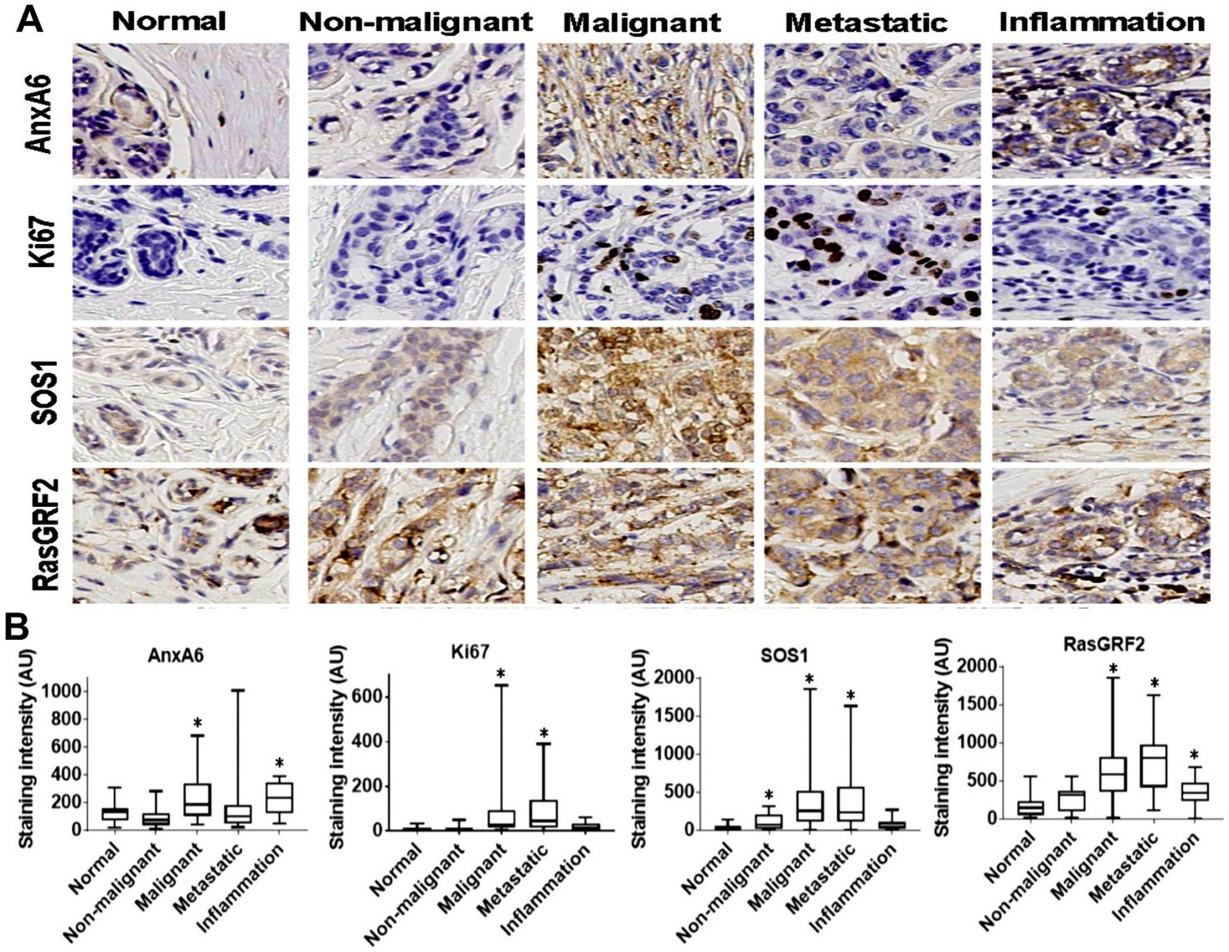
Expression of AnxA6 and proliferation markers in normal breast and breast disease tissues. A) Thin sections of formalin-fixed paraffin-embedded tissues in a broad-spectrum breast disease TMA were stained with antibodies against the indicated proteins. Shown are representative stained tissues. B) The stained tissues were digitally scanned and digitally analyzed using the Tissue IA software. Shown are boxplots depicting the mean staining intensity and distribution of AnxA6, Ki67, SOS1 and GRF2 staining intensity in the normal and the variety of breast disease tissues. * indicates p<0.05 for relative staining intensity of each protein in the indicated subgroup of breast disease tissues compared to normal breast tissues.

We previously reported that high AnxA6 and low RasGRF2 expression is associated with poorer distant relapse-free survival of basal-like breast cancer patients compared to patients with low AnxA6 and high GRF2 expressing basal-like breast cancer. However, this relationship was not obvious when all breast cancer patients were considered as a single cohort [22]. Given that low AnxA6 expression is associated with rapid tumor cell proliferation [12] and early initiation and rapid growth of xenograft tumors *in vivo* [17], we sought to determine whether the expression levels of AnxA6, GRF2 or SOS1 were associated with each other and with Ki67 expression levels in a breast disease progression tissue microarray (TMA). To accomplish this, we stained normal and breast disease tissues in a TMA that included non-malignant fibroadenomas and hyperplasia, malignant mostly carcinomas, inflammation, and metastatic breast cancer tissues with antibodies against AnxA6, GRF2, SOS1 and Ki67 (Fig 2A). For each antibody, and as indicated in materials and methods, the staining was initially validated with established positive control tissues at the Vanderbilt Translational Pathology Shared Resource (TPSR) prior to staining of the TMAs. As expected, AnxA6, GRF2 and SOS1 are mostly cytoplasmic proteins, while Ki67 is nuclear. The staining intensities of Ki67, SOS1 and GRF2 were significantly elevated (p<0.05) in malignant and metastatic breast cancer tissues compared to normal tissues (Fig 2B). We also show that the expression of SOS1 was increased (p = 0.039) in non-malignant tissues, while the expression of GRF2 was higher (p = 0.011) in inflamed breast tissues (Fig 2A and 2B). Compared to normal tissues, the expression levels of AnxA6 were relatively higher in malignant (p = 0.010) and inflamed (p = 0.017) breast tissues but were unchanged in metastatic tissues (Fig 2B). Given that Ki67 is a marker for cell growth, and that GRF2 and SOS1 promote cell growth via activation of Ras proteins, the increased expression of these proteins in malignant breast and metastatic tumors is consistent with the relatively rapid growth of these tumors compared to normal tissue. These data also highlight subtle differences in the involvement of different RasGEFs in non-malignant tissues and during inflammation of breast tissues.

**Fig 3:**
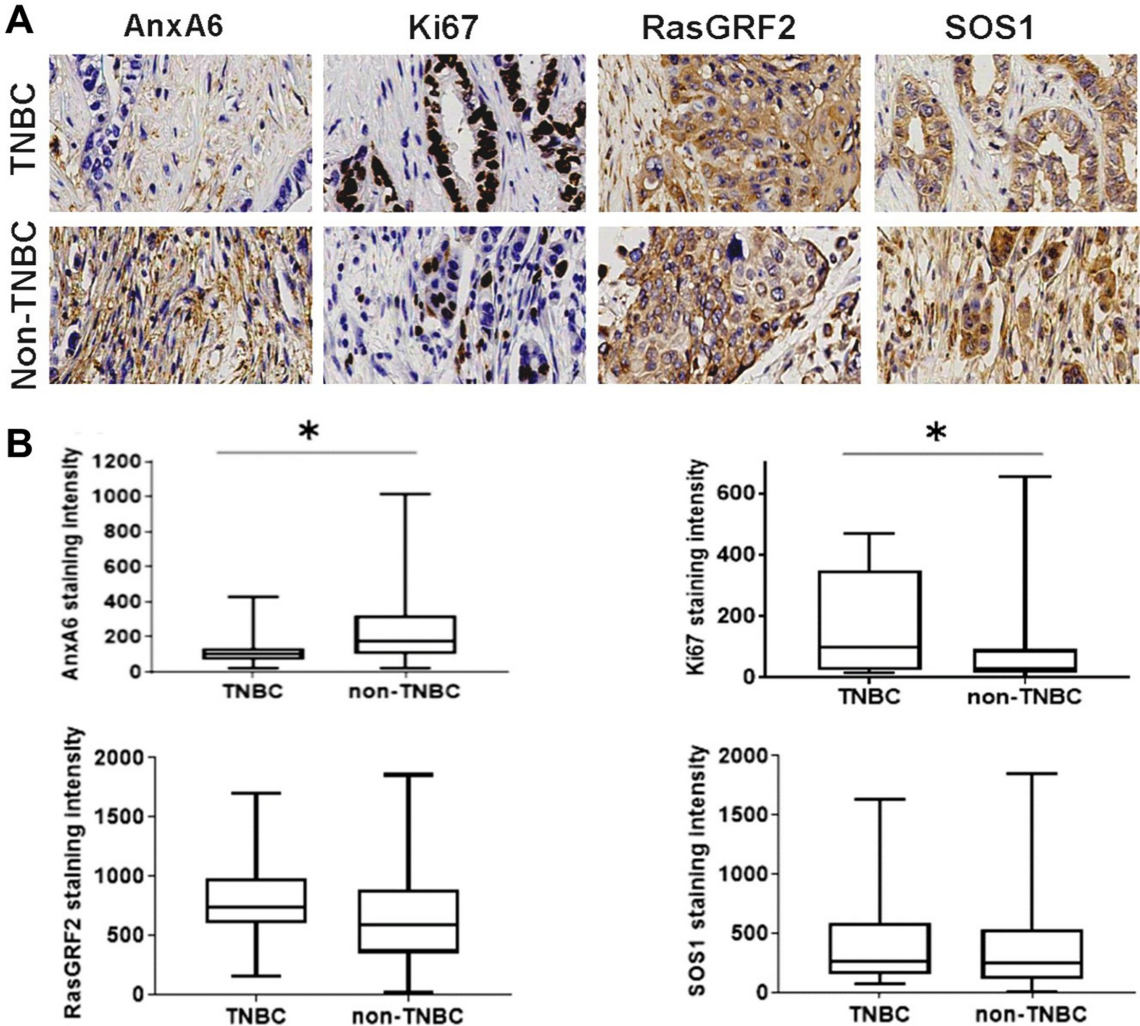
Expression of AnxA6 and proliferation markers in malignant and metastasis breast cancer tissues. Tissues were processed as described in Fig 2 and the malignant and metastasis tissues stratified into TNBC and non-TNBC subsets. Shown are representative stained tumor tissues (A) and analysis of the staining intensities of the indicated proteins by using the Tissues IA software (B). * indicates p<0.05 for the mean staining intensity of each protein in the TNBC subset compared to the non-TNBC subset.

Since the expression levels of AnxA6 increased with disease progression, we next evaluated whether AnxA6 expression levels detected by IHC differed between TNBC (n = 15) and non-TNBC (n = 116) malignant/metastatic tumors. In these advanced breast cancers and as anticipated, Ki67 expression levels were higher in the TNBC group compared to the non-TNBC group (p = 0.026). While GRF2 and SOS1 expression levels did not significantly differ between TNBC and non-TNBC subsets, AnxA6 levels were significantly (p = 0.022) lower in TNBC tumors versus non-TNBC tumors (Fig 3A and 3B). Interestingly, the reduced expression of AnxA6 in TNBC tended to be associated with relatively higher expression levels of GRF2 in TNBC while the relatively high AnxA6 levels in non-TNBC tumors appeared to be associated with relatively lower GRF2 levels in non-TNBC tumors (Fig 3B). These data suggest that reduced expression of AnxA6 and to some extent the relatively higher GRF2 expression levels are mostly associated with the more proliferative (higher Ki67 positivity) TNBC tumors, consistent with the reduced expression of AnxA6 in highly malignant cancers [16].

### Expression status of AnxA6 and proliferation markers in TNBC tissues before and after cytotoxic chemotherapy

**Fig 4:**
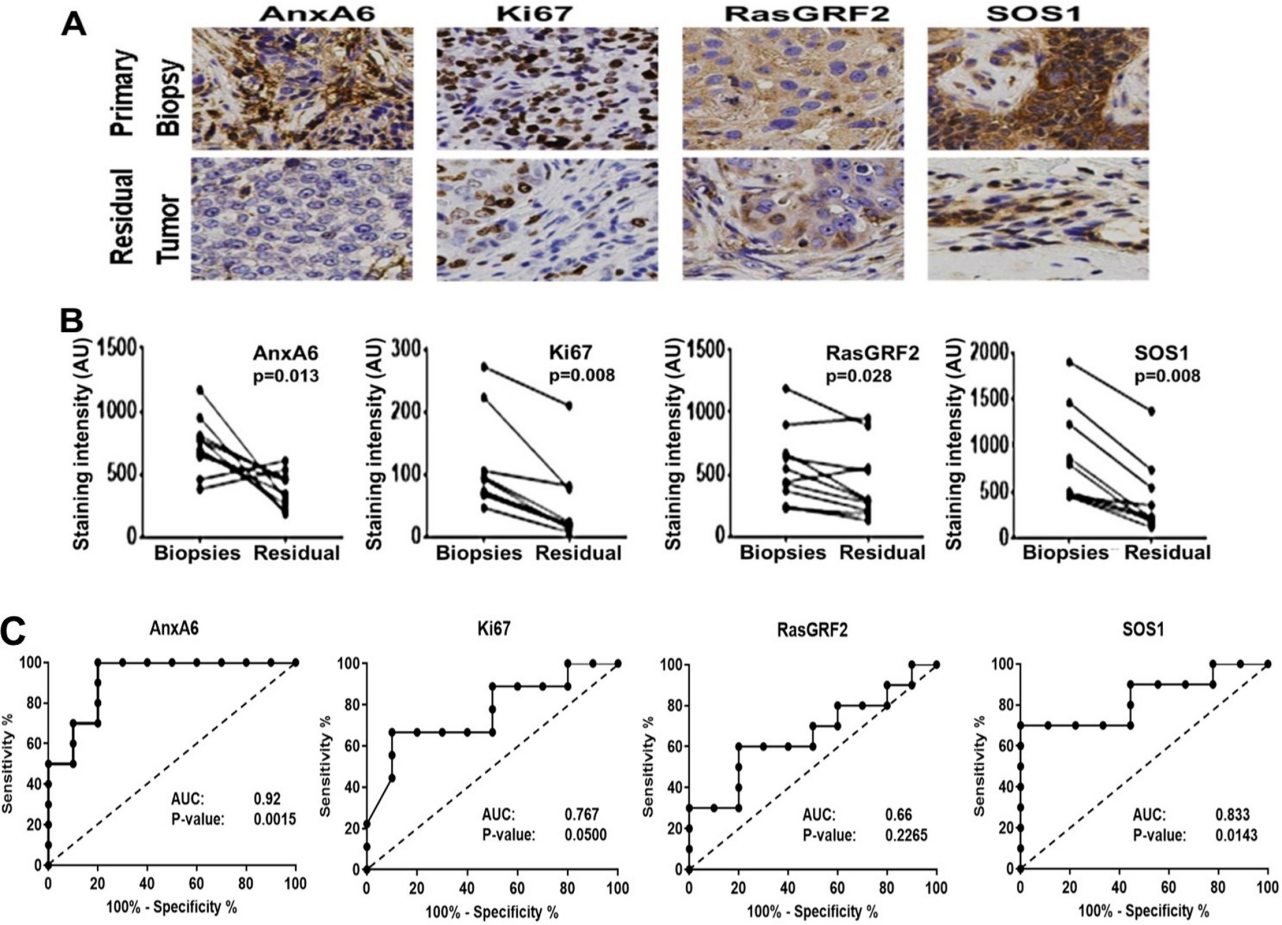
Expression status of AnxA6 and proliferation markers in response to cytotoxic chemotherapy. A) Formalin-fixed paraffin-embedded TNBC tissues from primary and residual tumors following chemotherapy were stained with antibodies against the indicated proteins. Slides were processed as in Fig 2, and representative stained tumor tissues. B) The staining intensity of the indicated proteins was quantified by using the Tissues IA software. The p-values represent the mean staining intensity of the respective proteins in the primary tumors (Biopsies) compared to that in the residual tumors (Residual). C) ROC assessment of the staining intensity of AnxA6 and other proliferation markers in biopsies from patients with residual disease versus the staining intensity in the matching residual tumors. AUC: area under the curve.

Since standard-of-care treatment for TNBC is combination chemotherapy, we wanted to assess the relationship between Ki67 positivity and the expression levels of AnxA6, GRF2 and SOS1 in TNBC tissues from 22 TNBC patients who were treated with neoadjuvant chemotherapy (Table 1). To accomplish this, we stained both pre-treatment biopsies and where applicable, the residual disease tissues by IHC with antibodies against AnxA6, Ki67, GRF2, and SOS1. Following the neoadjuvant chemotherapy treatment, 10 patients (45.5%) showed no residual tumors (NRT) while 12 patients (54.5%) had residual tumors (RT) with tumor sizes ranging from 0.5 to 37 mm (Table 1). AnxA6, Ki67, GRF2, and SOS1 expression levels were significantly lower in the residual chemotherapy-resistant tumors compared to primary pre-treatment biopsies from patient-matched tissue (Fig 4A and 4B). Given that GRF2 and SOS1 are activated by receptor tyrosine kinases such as EGFR, we stained the tissues with antibodies against total EGFR as well as phospho-EGFR (Y1068) and phospho-ERK1/2 that recognizes the activated form of EGFR and ERK1/2 respectively. This analysis revealed that the expression levels of total and activated EGFR were not significantly altered by chemotherapy, but downstream activation of the MAPK pathway was significantly decreased in the residual tumors as demonstrated by decreased phospho-ERK1/2 staining (S2 Fig) and consistent with data in Fig 4. The lower expression levels of Ki67, GRF2, SOS1 and phospho-ERK1/2 in the residual TNBC tumors are consistent with initial slow growth or dormancy of these TNBC tumors following chemotherapy. However, decreased AnxA6 expression in the residual TNBC tumors may suggest the potential for these tumors to eventually grow rapidly.

We next used Mann-Whitney test to assess whether the expression levels of AnxA6, GRF2, SOS1 or Ki67 were different in the primary biopsies from patients with residual tumors (n = 12) versus those without residual tumors (n = 10) post-neoadjuvant chemotherapy. This analysis showed that the expression levels of these markers were not significantly different between these two cohorts of tissues (S3A Fig). To determine whether the expression levels of Ki67, GRF2, SOS1 and AnxA6 in pre-treatment biopsies could predict residual disease, we preformed sensitivity and specificity analysis using receiver operating characteristic (ROC) analysis. Although Ki67 and SOS1 were able to distinguish pre-treatment tumors that would eventually become resistant to therapy with residual disease, AnxA6 expression was the most reliable biomarker with an area under the curve (AUC) of 0.92 and p = 0.0015) to distinguish biopsies of pre-treatment tumors from residual disease (Fig 4C).

Due to the limited sample size of patient biopsies and matched residual tumors, we performed additional analysis comparing AnxA6, GRF2, SOS1 and Ki67 mRNA expression in a dataset containing 196 patients that responded to chemotherapy by achieving a pathological complete response and 277 patients who did not respond (non-responders) [21]. We demonstrate that TNBC patients expressing relatively higher levels of AnxA6 (AUC = 0.613, p = 8.1E-06) and GRF2 (AUC = 0.629, p = 0.012) had a greater likelihood of responding to chemotherapy (S3B Fig). Meanwhile, the expression levels of Ki67 were not different in TNBC tumors from responders and non-responders and ROC analysis revealed that Ki67 (AUC = 0.526, p = 0.17) is not a reliable biomarker for response of TNBC to chemotherapy (S3B Fig). Therefore, even though our sample size for chemotherapy treated TNBC patients was small, these data together suggest that AnxA6 is a more reliable biomarker to predict response to chemotherapy and TNBC relapse compared to the histopathology biomarker Ki67.

### The expression status of AnxA6 is associated with the survival of chemotherapy treated TNBC patients

Since AnxA6 expression has the potential to predict chemotherapy response, we next sought to determine the relationship between AnxA6, GRF2, SOS1 and Ki67 protein expression in the primary and residual tumors by Pearson correlation analysis. We found that the expression levels of Ki67, GRF2 and SOS1 were strongly and significantly associated with each other in primary tumors (n = 22) and with residual tumor size (n = 12) (Table 2). However, AnxA6 expression levels only moderately correlated with residual tumors size, and did not correlate with GRF2, Ki67 or SOS1 expression levels. Interestingly, EGFR expression also strongly correlated with the residual tumor size, suggesting that activation of RasGEFs might directly or indirectly define the growth of a subset of TNBC tumors after chemotherapy.

**Table 2:** Pearson correlations between the expression levels of proliferation markers in biopsies and residual tumors.

Markers	Pearson Correlation			
	Biopsies		Residual Tumors	
	Correlation (r)	p-value	Correlation (r)	p-value
Ki67 vs GRF2	0.8869	0.0006*	0.7435	0.0217*
Ki67 vs EGFR	0.7336	0.0157*	0.7843	0.0123*
Ki67 vs SOS1	0.8470	0.0040*	0.4605	0.2122
GRF2 vs EGFR	0.7105	0.0213*	0.8207	0.0036*
GRF2 vs SOS1	0.9489	0.0001*	0.8131	0.0042
SOS1 vs EGFR	0.6875	0.0407*	0.4447	0.1978
GRF2 vs size	NA	NA	0.8334	0.0027*
Ki67 vs size	NA	NA	0.4749	0.1654
AnxA6 vs size	NA	NA	0.5095	0.1325
SOS1 vs size	NA	NA	0.6079	0.0623
EGFR vs size	NA	NA	0.7819	0.0075*

NA: not applicable

* p<0.05.

**Fig 5:**
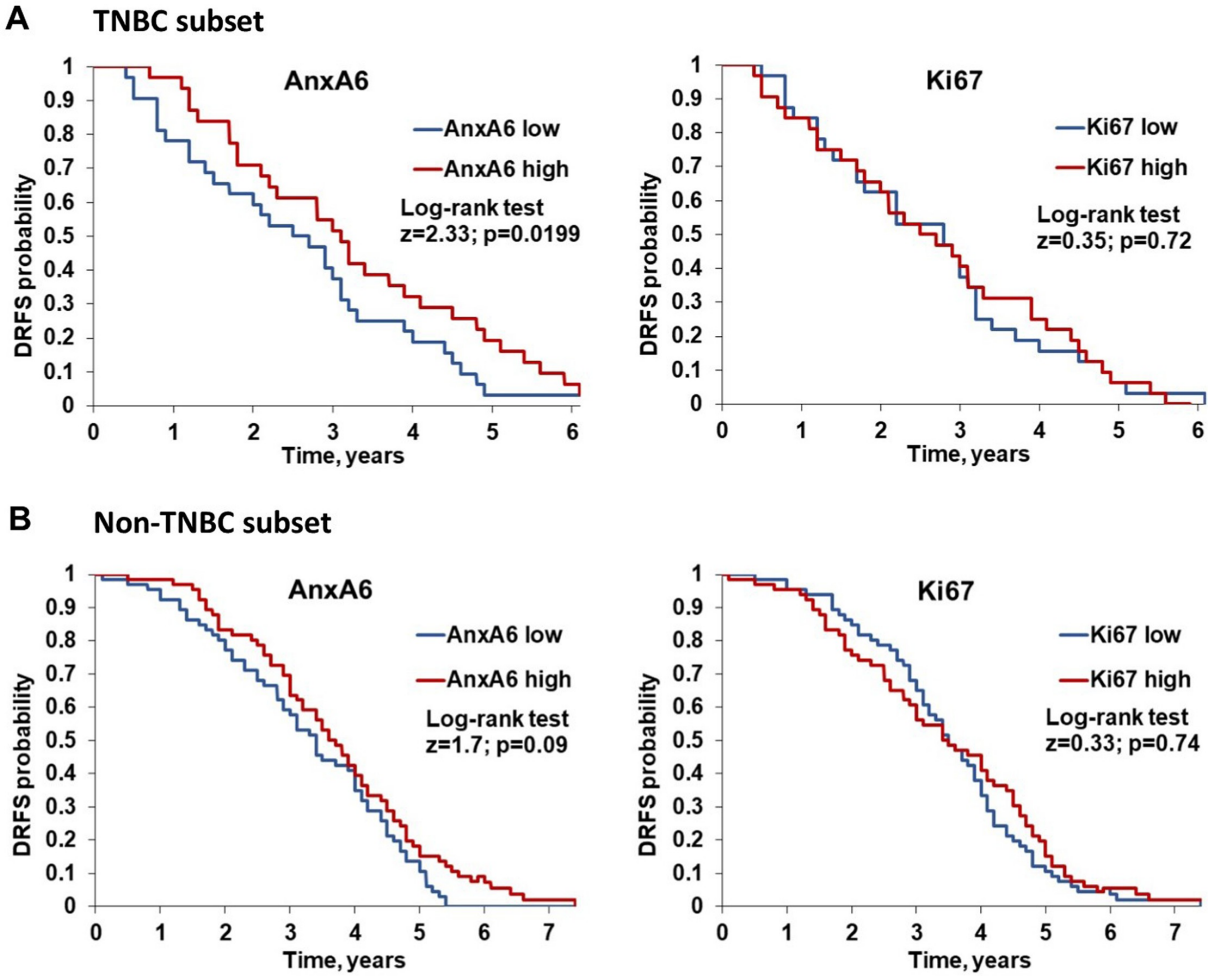
Association of the expression status of AnxA6 and Ki67 with distant relapse free survival of breast cancer patients following chemotherapy. Kaplan-Meier plots showing the relationship between high (red) and low (blue) AnxA6 or Ki67 expression and distant relapse free (DRFS) survival of TNBC patients (A) and non-TNBC patients (B).

We next evaluated the prognostic value of AnxA6 and Ki67 expression on distant relapse-free survival (DRFS) of TNBC and non-TNBC patients. To do this, we analyzed a dataset (GSE25065) containing 64 TNBC and 132 non-TNBC patients who underwent neoadjuvant taxane and anthracycline-based chemotherapy [20]. This analysis revealed that patients with low AnxA6-expressing TNBC tumors have a significantly shorter DRFS (p = 0.019) than patients with high AnxA6-expressing TNBC tumors (Fig 5A). In contrast, AnxA6 expression levels were not associated with DRFS in non-TNBC patients (Fig 5A). Interestingly, Ki67 expression was not associated with DRFS of either TNBC or non-TNBC patients (Fig 5B). A similar analysis for relapse free survival (RFS) of basal-like breast cancer patients using the KM plotter software for breast cancer [27] revealed that the expression level of GRF2 in basal-like breast tumors is significantly associated with relapse free survival (logrank P = 0.00098), while that of SOS1 is not. This analysis also revealed that the expression of low AnxA6/high GRF2 and high Ki67 is associated with poorer RFS of basal-like breast cancer patients compared to those expressing high AnxA6/low GRF2 and low Ki67 (S4 Fig). Together, these data not only suggest that low AnxA6 expression is associated with more aggressive TNBCs but also demonstrate the prognostic value of AnxA6 as an independent predictor of the response to chemotherapy and subsequent relapse of therapy resistant TNBC.

### The reciprocal expression of AnxA6 and GRF2 delineates rapidly growing basal-like from invasive mesenchymal-like TNBCs

**Fig 6:**
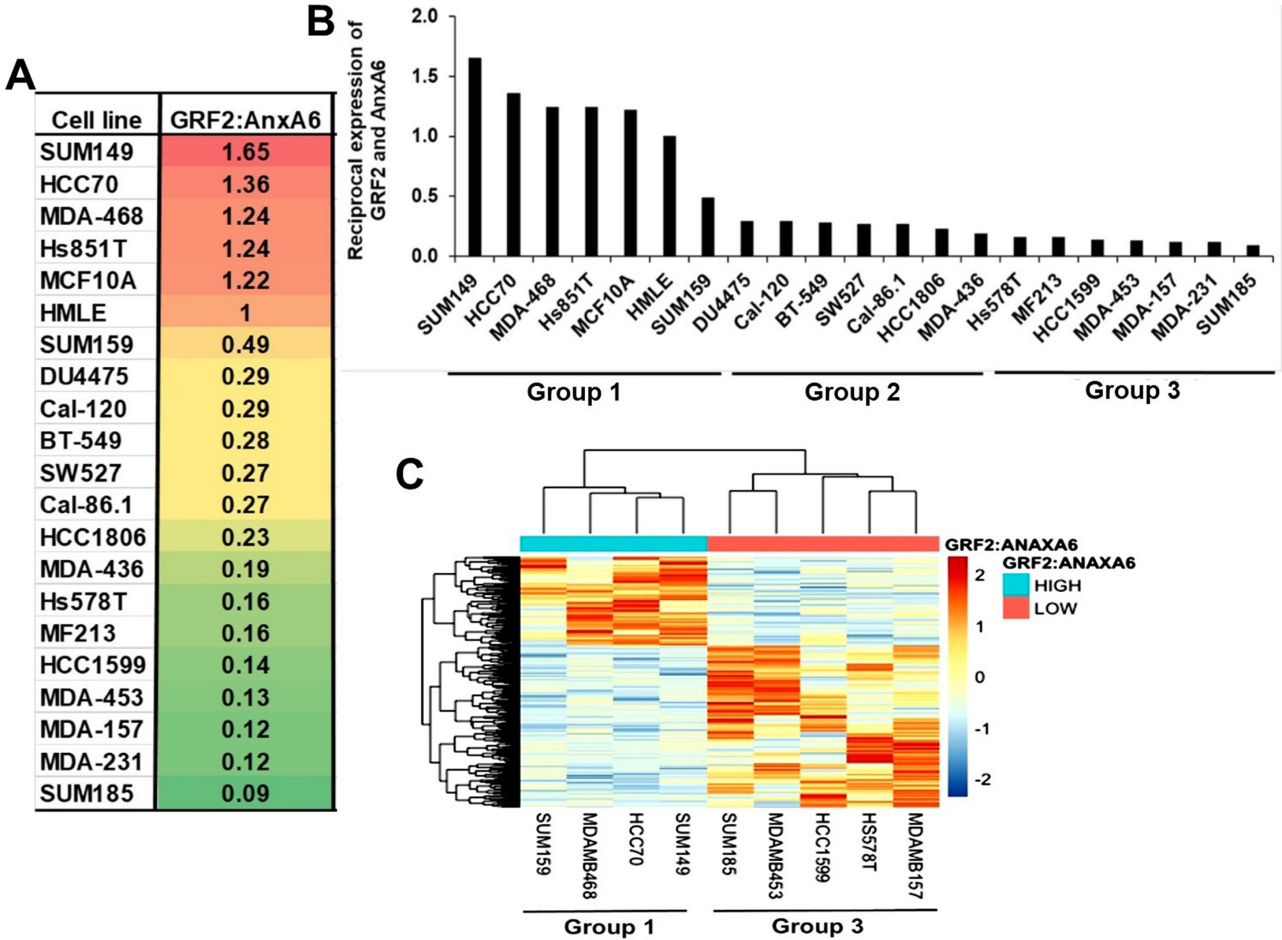
The reciprocal expression of AnxA6 and GRF2 delineates rapidly growing basal-like from invasive mesenchymal-like TNBC cell lines. A) Whole cell extracts were prepared from TNBC cell lines (n = 19) and normal breast epithelial cells (n = 2). The blots were analyzed by western blotting and densitometry as recently reported in Whalen et al., 2019 (Ref. 17). The ratio of GRF2:AnxA6 was used to stratify the cell lines according to their potential for rapid growth or invasiveness. B) A plot of the reciprocal expression of AnxA6 and GRF2 delineating TNBC cells with a higher potential for rapid growth (low AnxA6/high GRF2) from those with a higher potential for invasiveness (high AnxA6/low GRF2). C) A heat map of cells from Group 1 (n = 4) and Group 3 (n = 5) showing the clustering of the TNBC cell lines from each group and the discernible differences in their gene expression profiles.

Like most genes and their products, AnxA6 and GRF2 are heterogeneously expressed in TNBC cell lines [17] and in TNBC tumors as depicted by the variations in Figs 2 and 3. However, our data suggesting that low AnxA6 and high GRF2 expression levels are associated with poorer RFS of basal-like breast cancer patients compared to high AnxA6 and low GRF2 expression levels led us to speculate that the ratio of GRF2 to AnxA6 can delineate rapidly growing from invasive TNBCs. To test this we determined the ratio of GAPDH normalized intensity of GRF2 to AnxA6 protein bands in the immunoblot reported previously [17]. This analysis revealed that TNBC cells with basal-like morphology (ATCC, TCP-1001), herein designated group 1, including MDA-MB-468, HCC70 as well as other basal-like TNBC cell lines (SUM149 and Hs851T) are those with a GRF2:AnxA6 ratio (>1.0). On the contrary, most TNBC cells with mesenchymal and luminal morphology (ATCC, TCP-1002), herein designated group 3 are those with a low (<0.2) GRF2:AnxA6 ratio (Fig 6A). These include BT-549, Hs 578T, MDA-MB-231, MDA-MB-436, MDA-MB-157 and MDA-MB-453 (Fig 6A). Besides these two extreme groups of TNBCs, several other TNBC cell lines herein designated group 2, with a GRF2:AnxA6 ratio >0.2 but <1.0. (Fig 6A and 6B) comprises both basal-like (e.g. HCC1806) and mesenchymal-like (e.g. BT-549) TNBC cell lines. To validate the delineation of TNBC cells into those with a higher propensity for growth (GRF2-high/AnxA6-low) from those with a higher propensity for invasiveness, we compared the gene expression profiles of representative TNBC cell lines in group 1 (n = 4) and in group 3 (n = 5). For this analysis we selected SUM149, SUM159, MDA-MB-468 and HCC70 as representative Group 1 TNBC cell lines and SUM185, MDA-MB-543, HCC1599, Hs578T and MDA-MB-157 as representative Group 3 TNBC cell lines. As depicted in Fig 6C, the TNBC cell lines from each group clustered together and the gene expression profiles of group 1 cell lines are distinct from those from group 3 cell lines consistent with distinct gene expression profiles for basal-like and mesenchymal-like TNBC cell lines [7].

**Fig 7:**
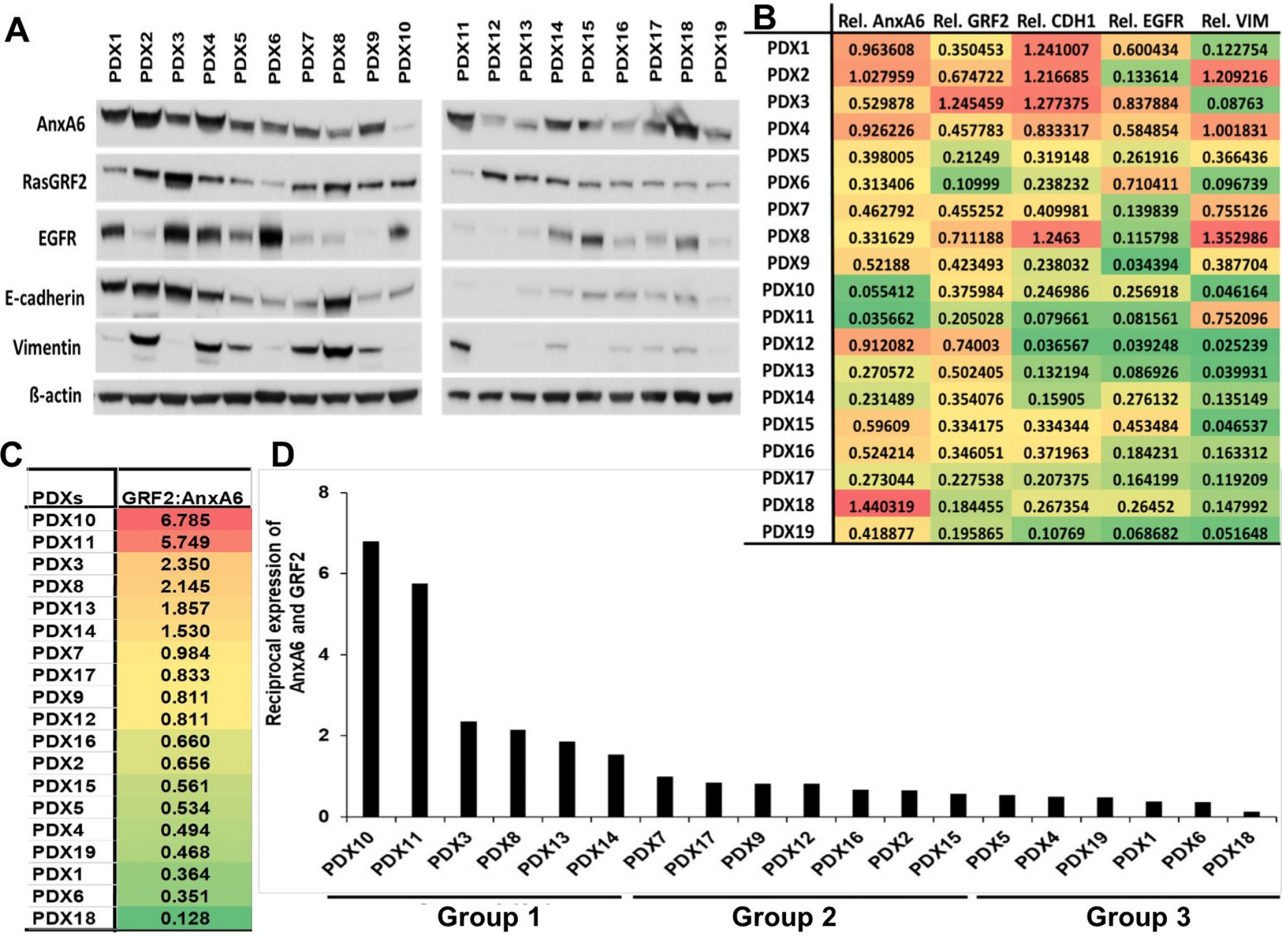
Reciprocal expression of AnxA6 and GRF2 in TNBC PDX models. A) Whole tissue extracts were prepared from patient derived xenografts (n = 19), and equal amounts of protein were analyzed by western blotting using antibodies to the indicated proteins. B) Densitometric analysis of the expression of the indicated proteins. Shown are protein band intensities normalized to β-actin from a representative experiment. C) The ratio of GRF2:AnxA6 was used to classify the tumors into potentially rapidly growing or invasive tumors as in Fig 6. D) A plot of the reciprocal expression of AnxA6 and GRF2 based on the GRF2:AnxA6 ratio for each PDX sample and showing two major groups: Group 1 with a strong potential for rapid growth and Group 3 with a strong potential for invasiveness, separated by a more diverse Group 2 with varied potentials for growth and invasiveness.

We then expanded our analysis to 19 patient derived xenografts (PDXs) and examined the expression of AnxA6, RasGRF2, EGFR, E-cadherin and Vimentin at the protein level (Fig 7A). Quantification of protein bands detected by these antibodies in the TNBC PDXs (Fig 7B) revealed heterogeneous expression of GRF2, AnxA6, as well as Vimentin, EGFR and E-cadherin (Fig 7A and 7B). Interestingly, like TNBC cell lines, the TNBC PDX tumors can also be grouped into potentially rapidly growing GRF2-high/AnxA6-low TNBC tumors with a GRF2:AnxA6 ratio >1.0 and potentially invasive GRF2-low/AnxA6-high TNBC tumors with a GRF2:AnxA6 ratio <0.5 (Fig 7C and 7D). The reciprocal expression of AnxA6 and GRF2 in TNBC PDXs also revealed three clearly discernible groups and effectively delineated those with a higher propensity for growth (GRF2-high/AnxA6-low) in group 1 from those with a higher propensity for invasiveness (GRF2-low/AnxA6-high) in group 3. All other TNBC PDXs designated as group 2, were those in which the expression of GRF2 and AnxA6 is less conspicuous and may not be mutually regulated (Fig 7D). Together, these data suggest that the reciprocal expression of GRF2 and AnxA6 in TNBC cell lines and PDX models can be used to select potentially rapidly growing mostly basal-like from the more invasive mostly mesenchymal-like TNBC cells and PDXs. Furthermore, this semi-quantitative representation of the reciprocal expression of AnxA6 and GRF2 is consistent with the intrinsic TNBC subtypes [7] and may constitute an inclusive TNBC subgrouping for further studies on the diverse responses of TNBC tumors to therapeutic interventions.

## Discussion

**Fig 8:**
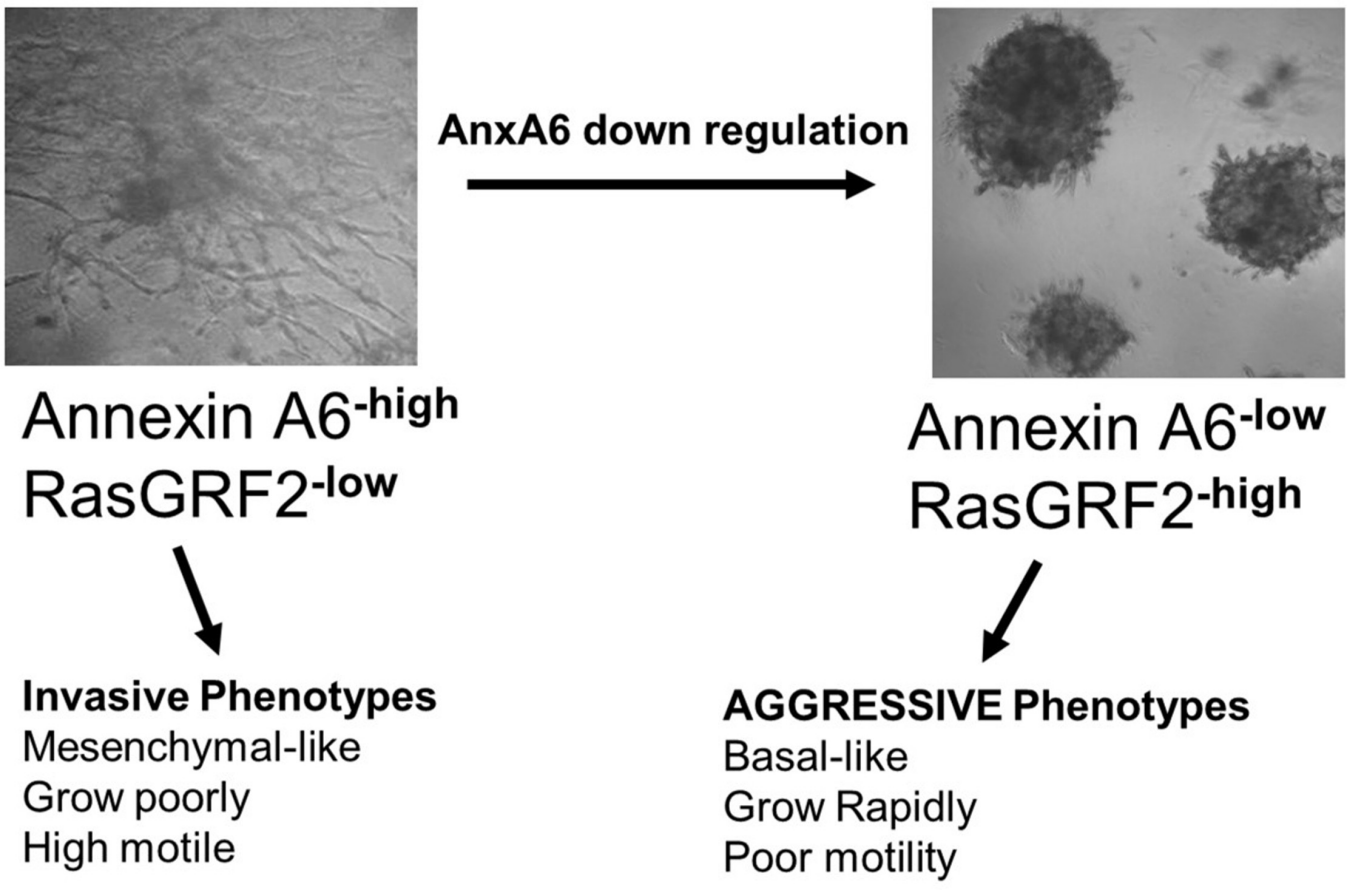
Distinct phenotypes of AnxA6 expressing and AnxA6 down regulated TNBC cells. Schematic showing 3D cultures in growth factor-reduced matrigel and the transformation of the AnxA6-high/GRF2-low invasive BT-549 TNBC cells into AnxA6-low/GRF2-high rapidly growing BT-549 TNBC cells following down regulation of AnxA6. It should be noted that parental BT-549 cells are poorly tumorigenic while AnxA6 depletion or loss in BT-549 leads to high tumorigenicity (see Ref. 17).

Breast cancer can be broadly classified into TNBC subgroup without targeted therapies and non-TNBC subgroup with targeted therapies against the ER, PR and/or HER2. These broad subgroups are known to differ in their growth characteristics, response to therapies and overall prognosis of patients [28]. Like breast cancer in general, the TNBC subtype, is also known to consist of a spectrum of molecularly distinct subsets with distinct clinical and pathological features [7]. Therefore, even though the expression of activated ERK1/2 or AKT [29], and either Ki67 or PCNA are used as standard proliferation markers [30, 31], assessment of TNBC prognosis based on these biomarkers remains challenging due to its heterogeneity. Ki67 is commonly assessed as a biomarker for tumor aggressiveness, resistance to chemotherapy, and to validate the risk of residual disease by immunohistochemistry [30, 31]. However, the correlation of Ki67 index with disease free survival has only been consistently shown in luminal A breast cancers [32]. In this study, we demonstrated that the reciprocal expression of AnxA6 and GRF2 is regulated by Ca^2+^ influx dynamics and that semi-quantitative analysis of the ratio of GRF2:AnxA6 can be used to broadly delineate the complex spectrum of TNBCs into rapidly growing and invasive subsets. As depicted in Fig 8, TNBC tumors expressing reduced levels of AnxA6 express higher levels of GRF2 and can be classified as rapidly growing tumors with high Ki67 positivity. On the contrary, TNBC tumors expressing high AnxA6 and low GRF2 can be classified as invasive tumors with low Ki67 positivity and proliferative potential. By delineating rapidly growing from invasive tumors, the GRF2:AnxA6 ratio may be useful as a complementary tool to the molecular classifications of TNBCs to evaluate the response of TNBC tumors to therapeutic interventions.

Although inhibition of non-selective Ca^2+^ channels suggest that the reciprocal expression of AnxA6 and GRF2 is dependent on inhibition of Ca^2+^ entry, the molecular process(es) and/or signaling pathway(s) remain to be completely elucidated. We recently showed that activation of oncogenic receptor tyrosine kinases (RTKs) e.g. EGFR, leads to Ca^2+^ influx dependent GRF2 activation and subsequent degradation, and that overexpression of AnxA6 blocks the activation/degradation of GRF2 [17]. This suggests that the degradation of GRF2 via proteasomes soon after its activation [33] is downstream of cell surface receptor-activated, AnxA6-modulated Ca^2+^ influx. It has also been shown that the expression of AnxA6 and GRF2 are epigenetically modulated in some cancers [13, 34], and that inhibition of Ca^2+^ signaling influences the expression of tumor suppressor genes via epigenetic mechanisms [35]. As a Ca^2+^ mobilizing receptor, we recently showed that inhibition of EGFR by chronic treatment of TNBC cells with tyrosine kinase inhibitors (TKIs) was associated with up regulation of AnxA6 [36]. Interestingly, a recent report revealed that TKIs inhibit store-operated Ca^2+^ influx channels [37], thus providing additional evidence that inhibition of Ca^2+^ channels underlies at least in part the reciprocal expression of AnxA6 and GRF2. Together, these reports suggests that inhibition of RTKs and/or AnxA6-modulated store-operated Ca^2+^ influx channels support the reciprocal expression of AnxA6 and GRF2 in TNBC cells. However, further studies are warranted to identify the Ca^2+^ channel(s) and the epigenetic mechanism(s) linking AnxA6 expression to GRF2 expression.

One of the major findings from this study is the observation that low AnxA6 expression and higher Ki67 positivity are more relevant in TNBC than in non-TNBC phenotypes and that AnxA6 is a more reliable biomarker than Ki67 for response to chemotherapy and subsequent relapse of therapy resistant TNBC. This is consistent with our earlier reports showing that low AnxA6 expression is associated with poor overall survival of patients with basal-like breast tumors [22] and the reported reduced expression of AnxA6 in malignant forms of several solid tumor types [16]. This is also supported by previous reports showing that detection of AnxA6 may be useful in identifying minimal residual disease in B-lineage acute lymphoblastic leukemia [38], and to stratify non-invasive cervical intraepithelial neoplasia from invasive squamous cervical cancer [38]. Whether the expression status of AnxA6 in primary TNBC tumors is predictive of the efficacy of chemotherapy, however, needs to be further investigated.

Over the years, several studies have demonstrated that TNBC tumors are frequently diagnosed as higher grade, larger and more aggressive breast tumors with frequent lymph node involvement. In spite of a brief response to neoadjuvant chemotherapy, the relapse-free survival for a majority of TNBC patients is shorter than that for patients with other breast cancer subtypes [39]. Our data not only confirm this notion but also suggest that more aggressive TNBC tumors are those that express reduced levels of AnxA6 and higher Ki67 levels. High Ki67 has been shown to be associated with higher tumor grades, increased potential for relapse and metastases, and poorer overall survival of breast cancer patients [40]. These prognostic features associated with high Ki67 are similar to those attributed to reduced expression levels of AnxA6. However, compared to Ki67 positivity which is a commonly used histopathological marker for disease aggressiveness [30, 31], reduced expression of AnxA6 in tumor tissues appears to be a more reliable tool to assess treatment outcomes for TNBC patients and in particular the tendency for the tumors to relapse following chemotherapy.

Histological grade and Ki67 have been shown to be significantly associated with positivity to EGFR activating mutations or gene amplification [41] which are common oncogenic events in some cancers. Since activating EGFR and Ras mutations are rare in breast cancer, activation of Ras proteins in TNBC may be dependent on mostly wild type oncogenic receptor tyrosine kinases via RasGEFs such as SOS1 and GRF2. Although these RasGEFs are potential oncogenes [42] and are known to activate Ras proteins by distinct mechanisms, it remained unclear whether differential expression of SOS1 and GRF2 is relevant in breast cancer. Our data showing that higher expression of these RasGEFs was associated with malignant and metastatic breast cancers is consistent with the oncogene addiction model [43]. The observation that GRF2 is highly expressed in malignant and inflamed breast tissues while SOS1 is highly expressed in malignant as well as non-malignant breast tissues also suggest subtle differences in the involvement of these RasGEFs in breast diseases including breast cancer.

The association of basal-like TNBCs with rapidly growing and mesenchymal-like TNBC with invasive phenotypes is supported by several lines of evidence, including the strong association of basal-like phenotypes with up regulation of proliferation transcriptional signatures and neovascularization [7, 44]. We have also shown that reduced expression of AnxA6 is associated with increased Ras activity and TNBC cell growth but on the contrary, reduced Cdc42 activity and cell motility [17]. It has also been shown that GRF2 activates Ras proteins to promote cell growth but on the contrary, block the activation of Cdc42 or Rac1 to inhibit cell motility [18, 19]. Consistent with these observations, this study also shows that the relapse-free survival of patients with basal-like breast cancers expressing high AnxA6 or low GRF2 is significantly poorer than that for patients expressing low AnxA6 or high GRF2 [17]. Together this supports the notion that the rapid growth or aggressive TNBC phenotype is depicted by TNBC cells expressing high GRF2 and low AnxA6, while the invasive phenotype is depicted by GRF2-low and AnxA6-high expressing TNBC cells. It is also appears that TNBC cells and tumors in which the cellular levels of AnxA6 and GRF2 are invariable or are not mutually regulated may be those that neither grow rapidly nor are invasive.

## Conclusions

Overall, our data suggest that reduced expression of AnxA6 is more relevant in TNBC molecular subtypes and that detection of AnxA6 expression may be an independent biomarker for distant relapse-free survival of chemotherapy treated TNBC patients. This study also suggests that the reciprocal expression of AnxA6 and GRF2 is regulated by Ca^2+^ influx dynamics and can be used to broadly delineate the complex spectrum of TNBCs into rapidly growing and invasive subsets which may be more relevant for studies on therapeutic interventions.

## Supporting information

S1 Data Original uncropped and unaltered blot images. (PDF)

S1 Fig Effects of Ca2+ channel blockers on AnxA6 and GRF2 in normal breast and TNBC cells. (DOCX)

S2 Fig Expression of EGFR, p-EGFR and p-ERK1/2 in primary and residual TNBC tumors. (DOCX)

S3 Fig Prediction of response to cytotoxic chemotherapy based on the expression status of AnxA6, GRF2, SOS1 and Ki67 in TNBC tissues. (DOCX)

S4 Fig Relationship between the expression status of AnxA6, GRF2, SOS1 and Ki67 in TNBC tissues and survival of basal-like breast cancer patients. (DOCX)
