# Supplementary figures and images for: Reciprocal expression of Annexin A6 and RasGRF2 discriminates rapidly growing from invasive triple negative breast cancer subsets
Source: PLoS One. 2020 Apr 16;15(4):e0231711. doi: 10.1371/journal.pone.0231711 (PMC7162501; doi:10.1371/journal.pone.0231711)

Fig 1A

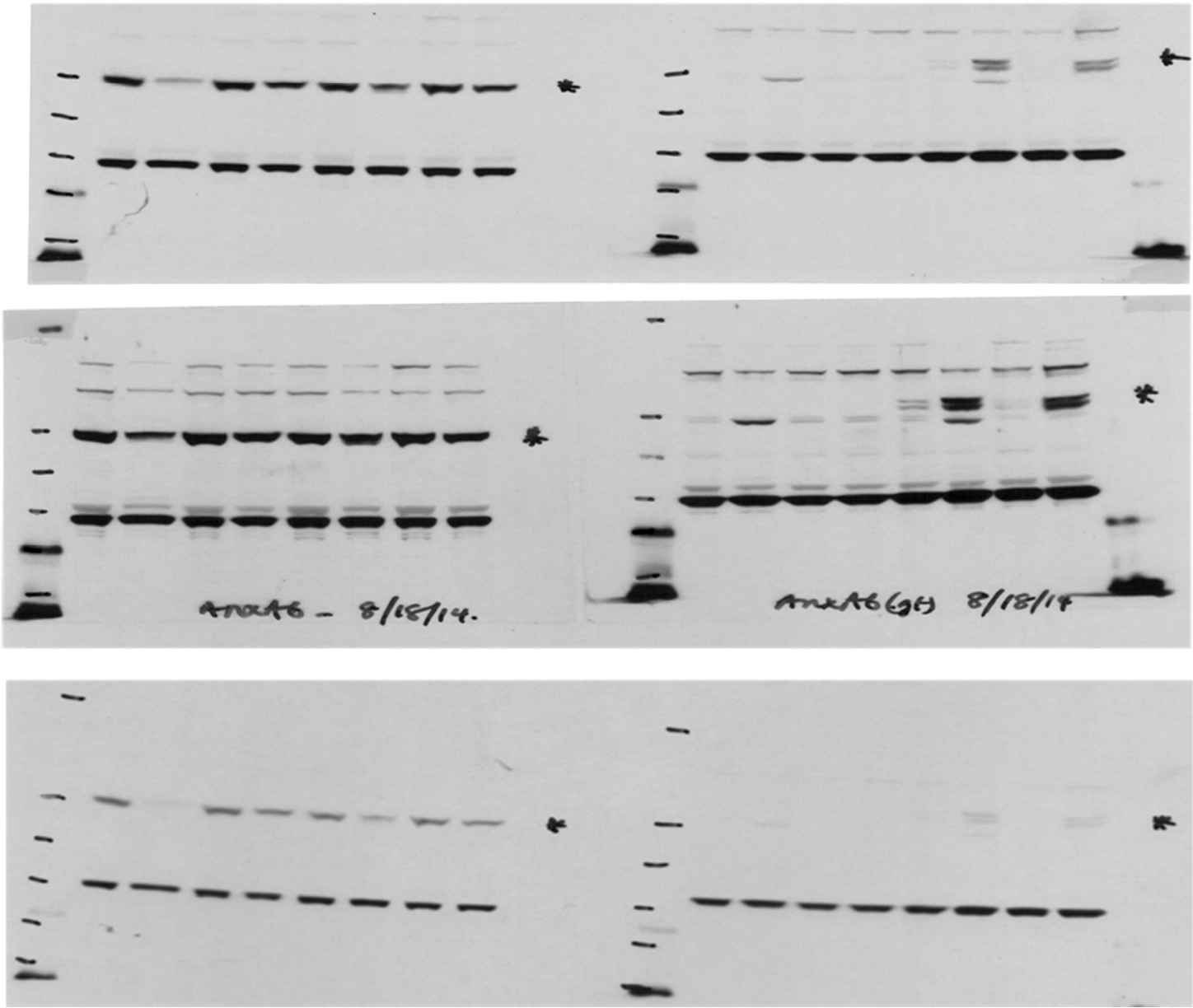

Fig 1C and D

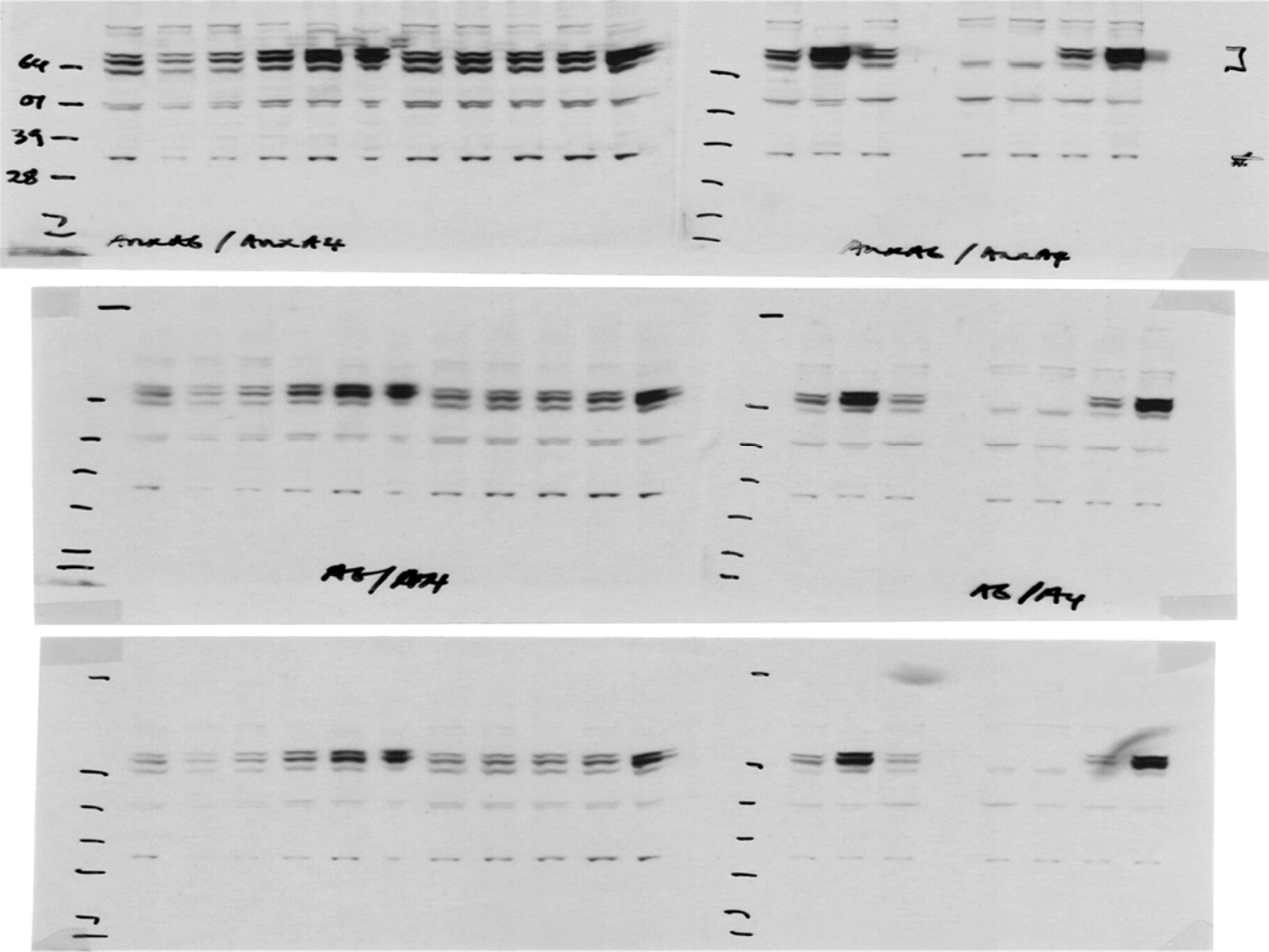

Fig 1C and D

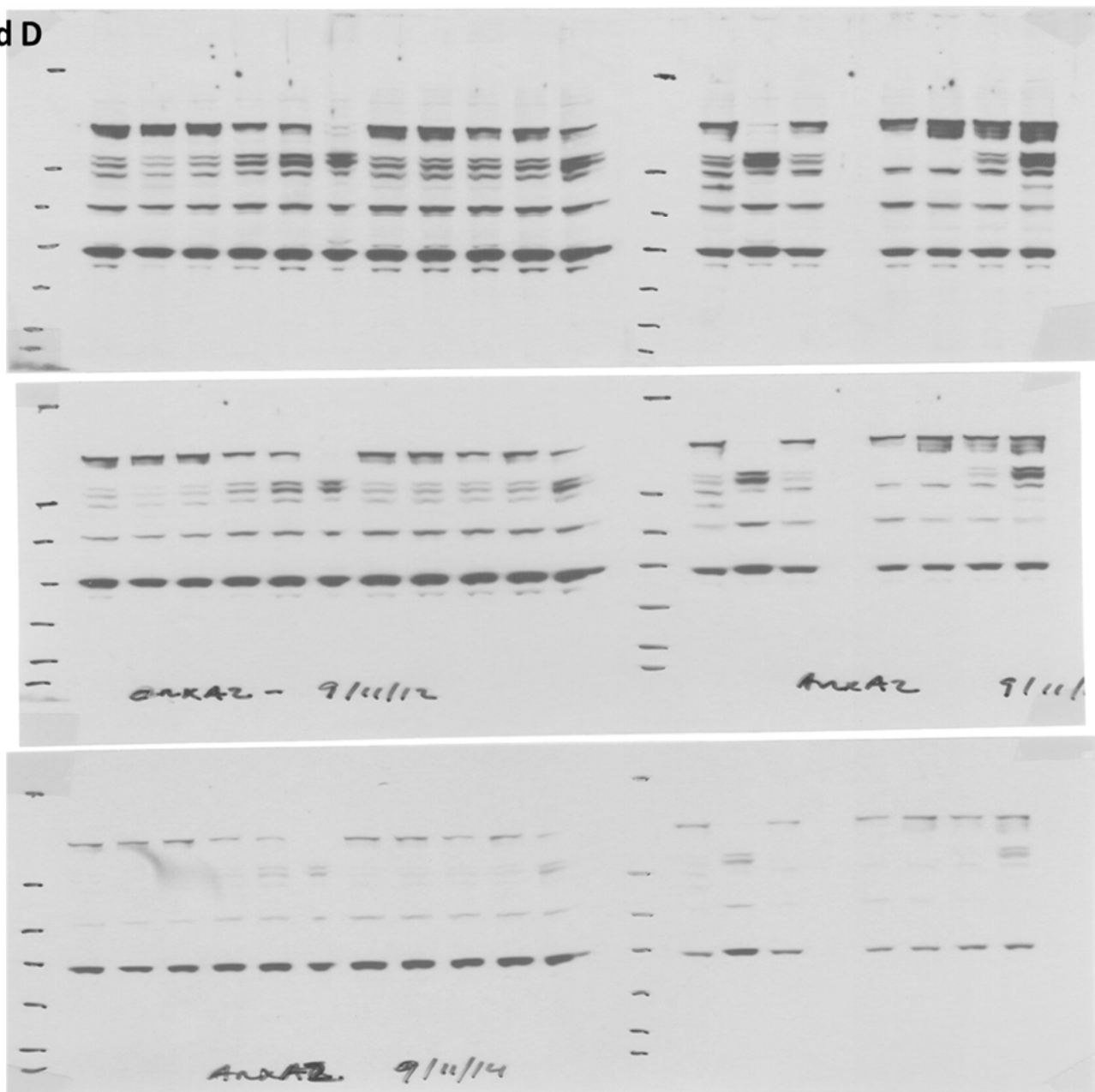

Fig 1E

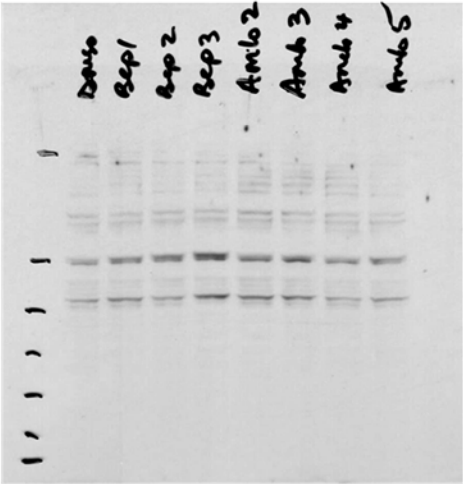

AnxA6

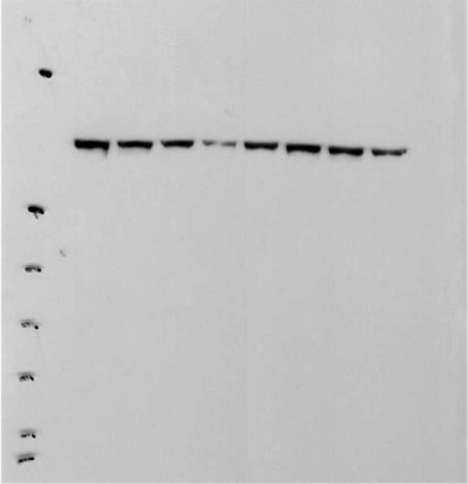

RasGRF2

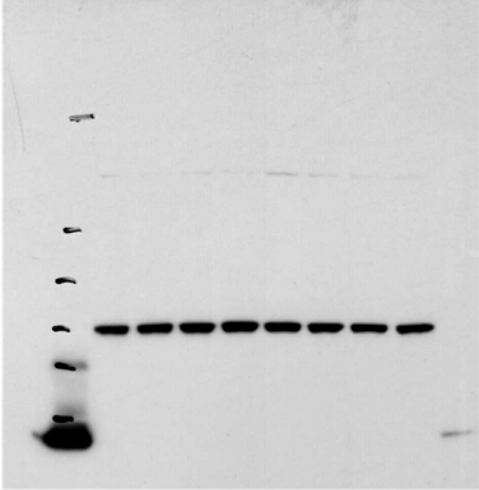

GAPDH

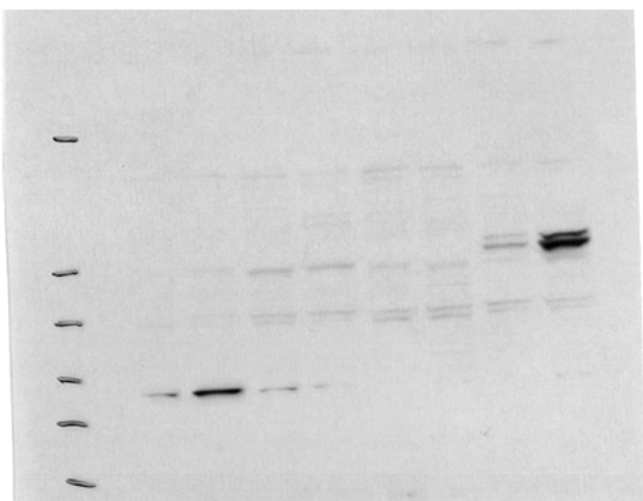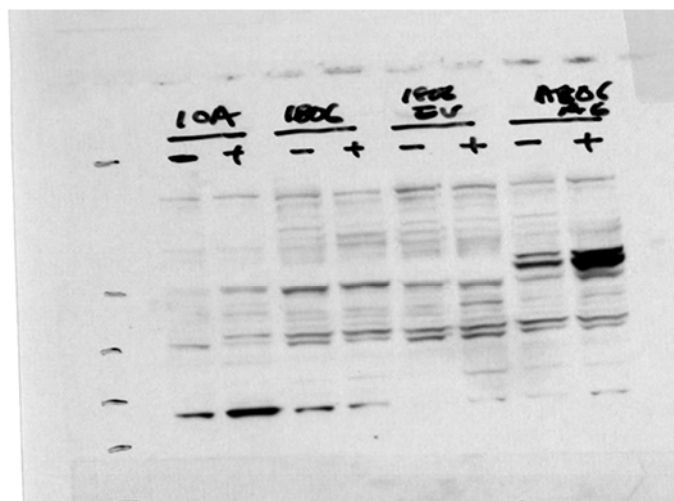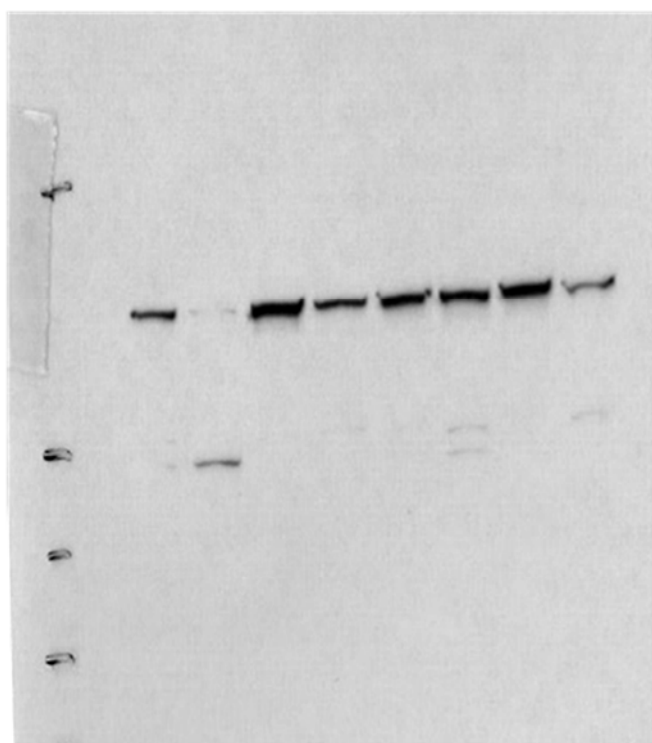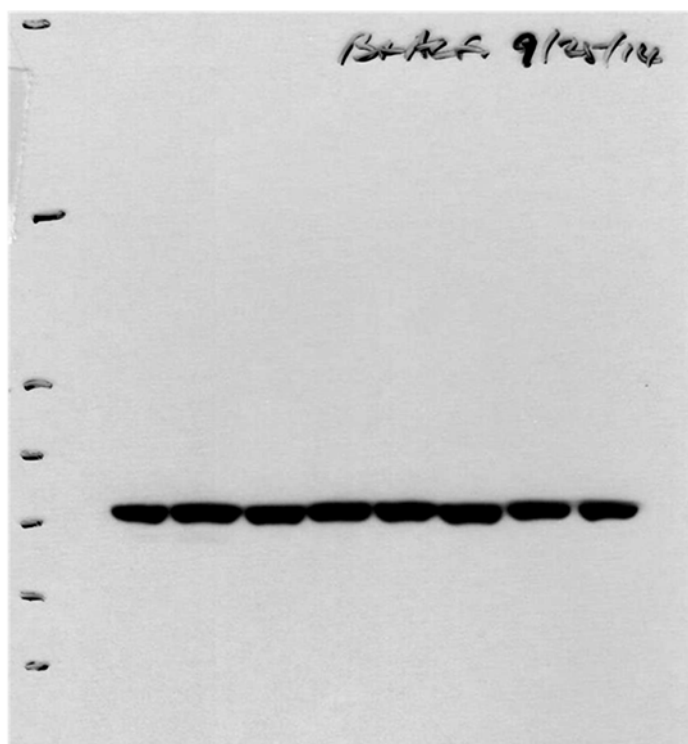

Supplementary Fig. S1A

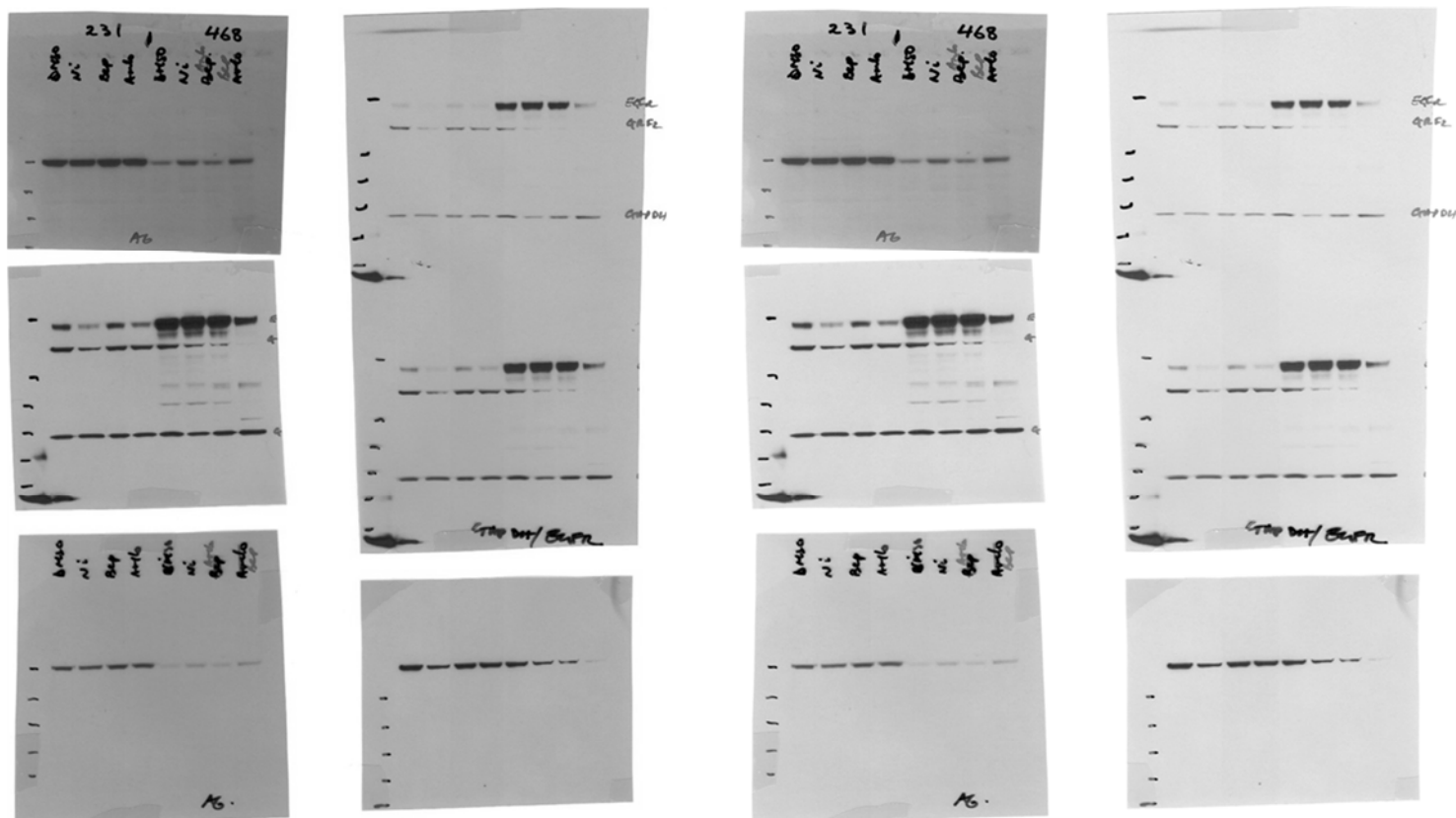

Supplementary Fig. S1B

Supplement: S1 Data — (PDF) [file pone.0231711.s001.pdf]
