# Supplementary material for: Reciprocal expression of Annexin A6 and RasGRF2 discriminates rapidly growing from invasive triple negative breast cancer subsets
Source: PLoS One. 2020 Apr 16;15(4):e0231711. doi: 10.1371/journal.pone.0231711 (PMC7162501; doi:10.1371/journal.pone.0231711)
Supplement: S1 Fig — (DOCX) [file pone.0231711.s002.docx]

**
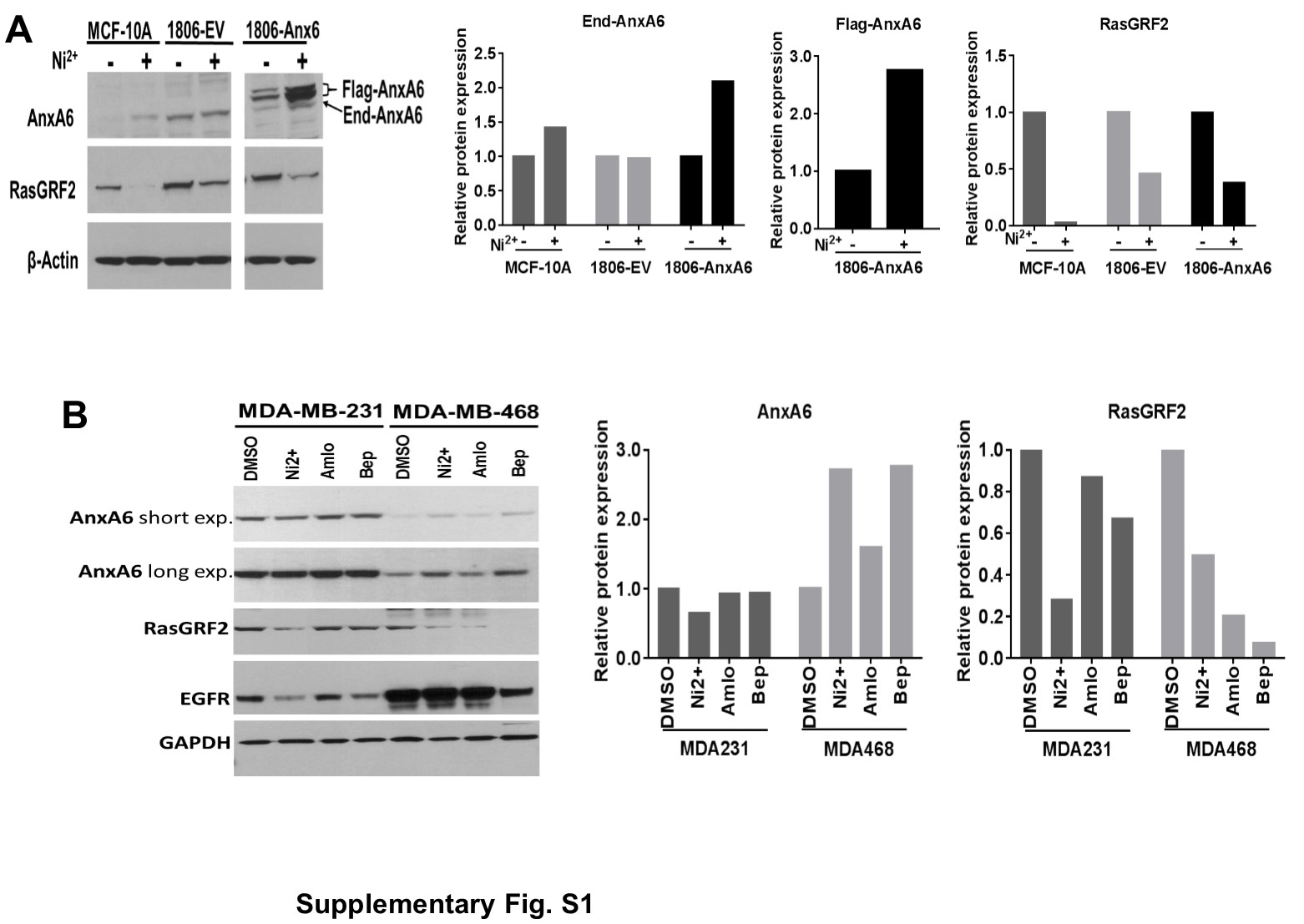
**

**Supplementary Fig S1. Effects of Ca^2+^ channel blockers on AnxA6 and GRF2 in normal breast and TNBC cells.** A) MCF10A and HCC1806 expressing the empty vector or flag-tagged AnxA6 were treated with or without Ni^2+^ and AnxA6 and RasGRF2 protein expression analyzed by western blotting. β-actin protein expression was used as the loading control. B) MDA-MB-231 and MDA-MB-468 cells were treated with the indicated compounds for 72 h and the expression of AnxA6, GRF2 and EGFR assessed by western blotting. Also shown are densitometric analysis of the expression of AnxA6 and GRF2 from a representative experiment (A and B, Left panels). GAPDH was used as the loading control. DMSO: dimethylsulfoxide; Amlo: Amlodipine; Bep: Bepredil.
