# Supplementary material for: Reciprocal expression of Annexin A6 and RasGRF2 discriminates rapidly growing from invasive triple negative breast cancer subsets
Source: PLoS One. 2020 Apr 16;15(4):e0231711. doi: 10.1371/journal.pone.0231711 (PMC7162501; doi:10.1371/journal.pone.0231711)
Supplement: S2 Fig — (DOCX) [file pone.0231711.s003.docx]

**
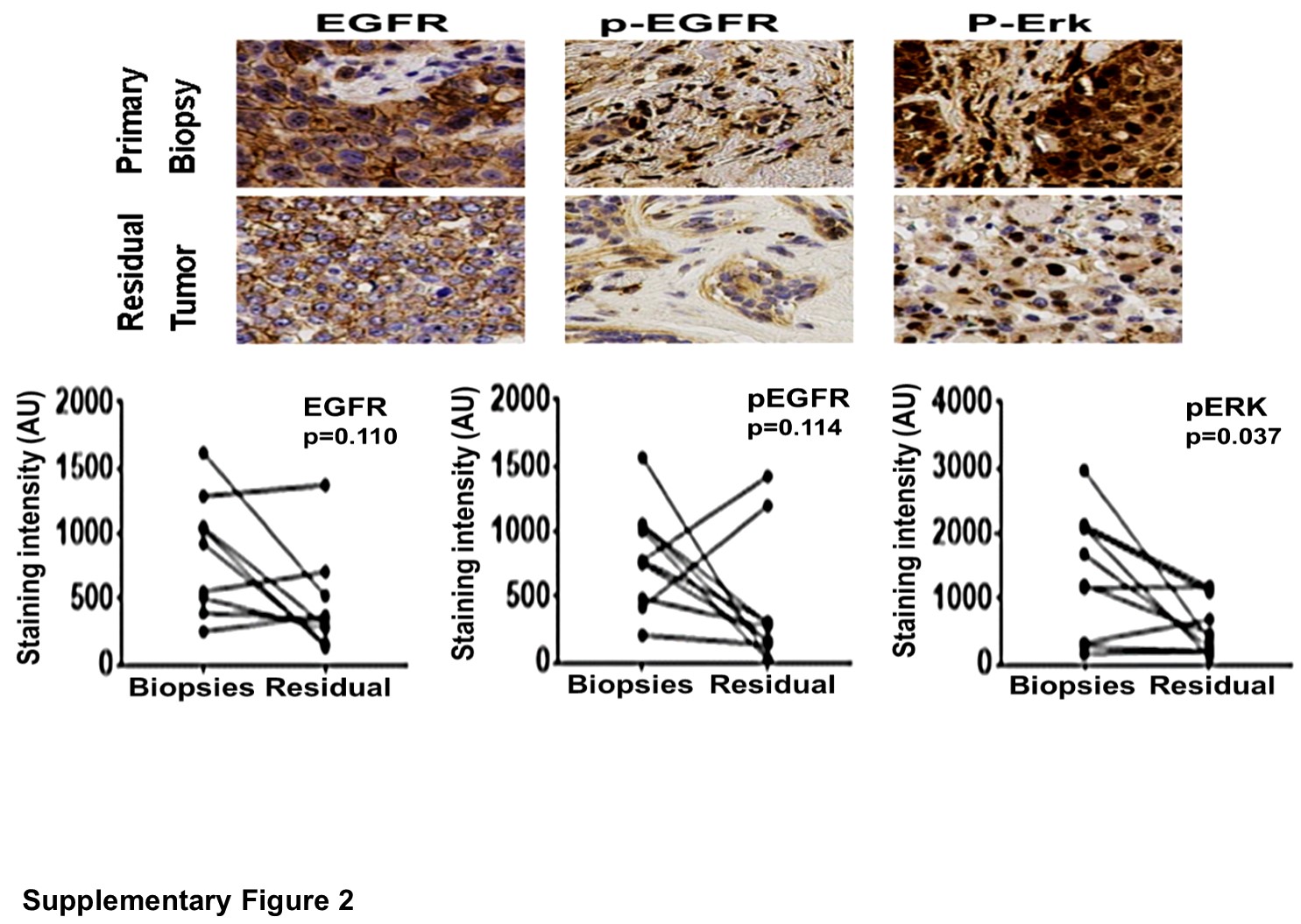
**

**Supplementary Fig S2. Expression of EGFR, p-EGFR and p-ERK1/2 in primary and residual TNBC tumors**. Primary pre-treatment and residual chemotherapy resistant tissues were processed by IHC and the expression of these proteins analyzed using the Tissues IA software. Indicated are comparisons of the relative expression of these proteins in the primary tumors versus the residual tumors.
