# Supplementary material for: Reciprocal expression of Annexin A6 and RasGRF2 discriminates rapidly growing from invasive triple negative breast cancer subsets
Source: PLoS One. 2020 Apr 16;15(4):e0231711. doi: 10.1371/journal.pone.0231711 (PMC7162501; doi:10.1371/journal.pone.0231711)
Supplement: S3 Fig — (DOCX) [file pone.0231711.s004.docx]

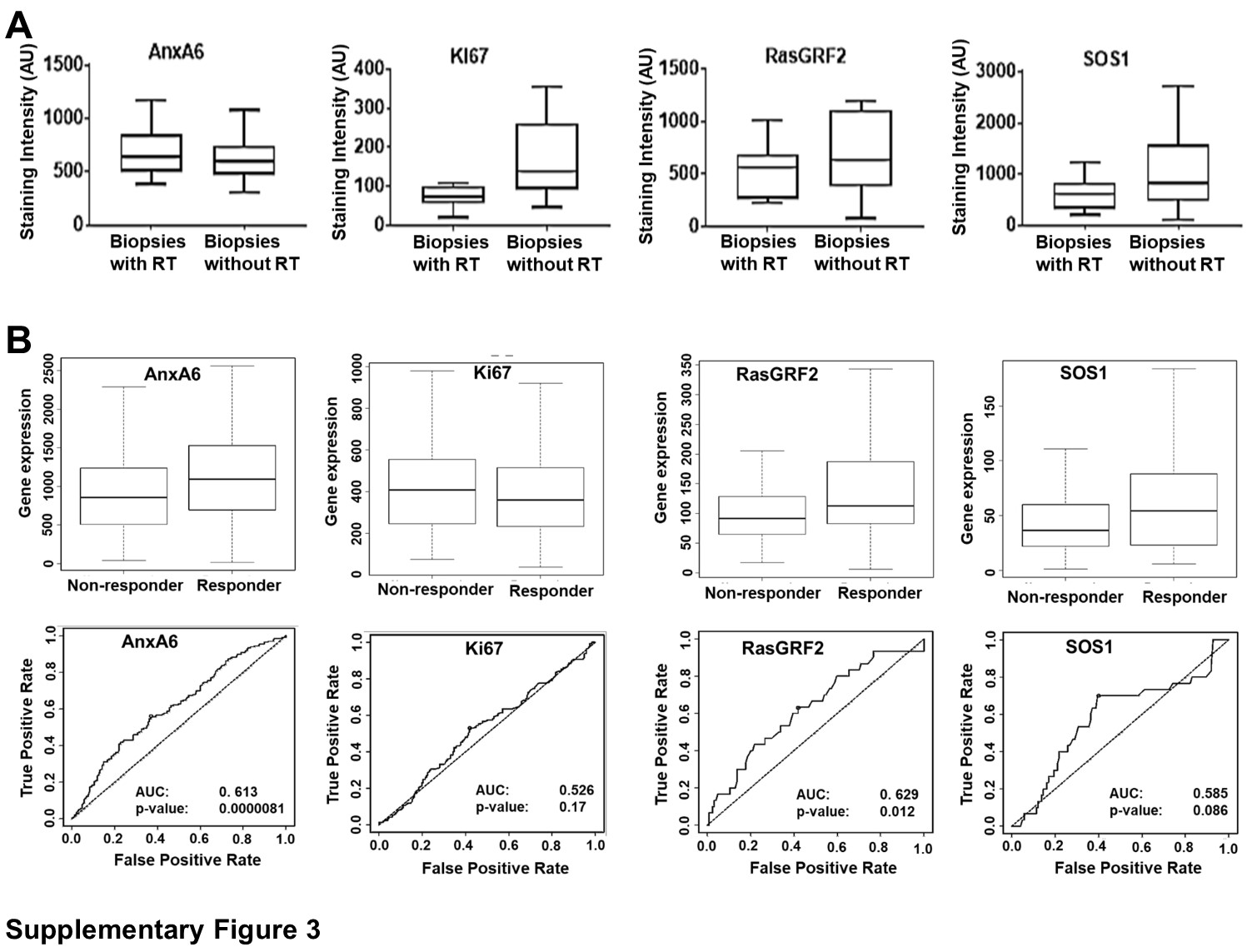


Supplementary Fig S3. Prediction of response to cytotoxic chemotherapy based on the expression status of AnxA6, GRF2, SOS1 and Ki67 in TNBC tissues. A) Thin sections of FFPE TNBC tumor tissues from chemotherapy treated patients with or without residual disease were stained with antibodies against the indicated proteins, and analyzed using the Tissue IA software. B) The ROC plotter was used to predict the likelihood of response to chemotherapy based on the expression status of AnxA6, GRF2, SOS1 and Ki67 in TNBC tissues. The dataset contained 196 patients who responded (Responders) and 277 patients who did not respond (Non-responders) to chemotherapy, based on pathological complete response. AUC: area under the curve.
