# Supplementary material for: Reciprocal expression of Annexin A6 and RasGRF2 discriminates rapidly growing from invasive triple negative breast cancer subsets
Source: PLoS One. 2020 Apr 16;15(4):e0231711. doi: 10.1371/journal.pone.0231711 (PMC7162501; doi:10.1371/journal.pone.0231711)
Supplement: S4 Fig — (DOCX) [file pone.0231711.s005.docx]

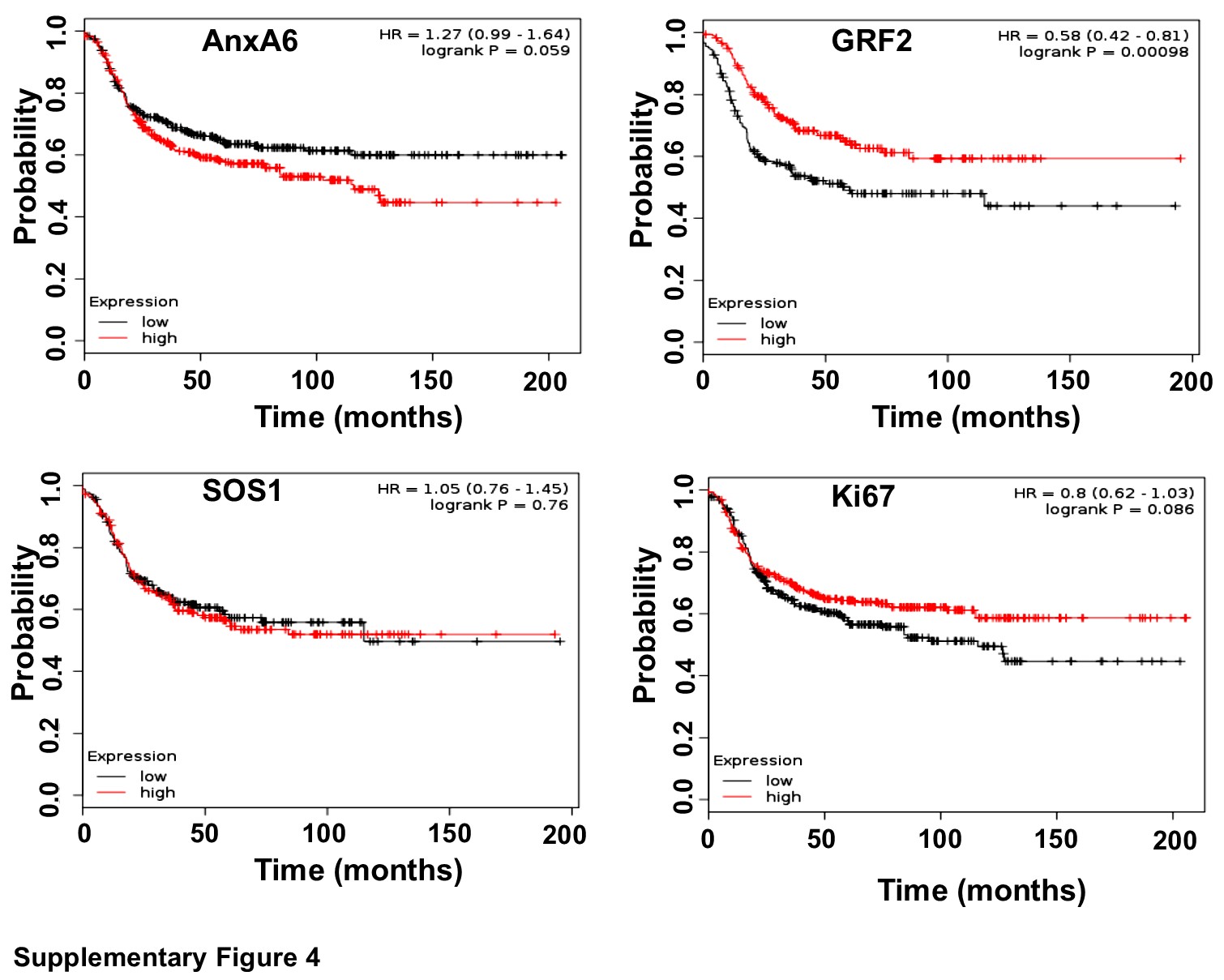


**Supplementary Fig S4**. **Relationship between the expression status of AnxA6, GRF2, SOS1 and Ki67 in TNBC tissues and survival of basal-like breast cancer patients.** Kaplan Meier plots showing the relationship between the expression of the indicated proteins and relapse-free (RFS) survival of basal-like breast cancer patients. The Kaplan-Meier plots are indicated for low gene expression status (black) and for high gene expression status (red).
